# Supplementary material for: Structural basis for malate-driven, pore lipid-regulated activation of the Arabidopsis vacuolar anion channel ALMT9
Source: Nat Commun. 2025 Feb 20;16:1817. doi: 10.1038/s41467-025-56940-5 (PMC11842843; doi:10.1038/s41467-025-56940-5)
Supplement: Supplementary file 1 — Supplementary Information [file 41467_2025_56940_MOESM1_ESM.pdf]

Supplementary Information

Structural basis for malate-driven, pore lipid-regulated activation of the Arabidopsis vacuolar anion channel ALMT9

Yeongmok Lee[1,†], Elsa Demes-Causse[2,†], Jaemin Yoo[3], Seo Young Jang[4], Seoyeon Jung[1], Justyna Jaślan[2], Geum-Sook Hwang[4,5], Jejoong Yoo[3], Alexis De Angeli[2] and Sangho Lee[1,6,7,*]

[1]Department of Biological Sciences, Sungkyunkwan University, Suwon 16419, Republic of Korea

[2]IPSiM, CNRS, INRAE, Institut Agro, Université Montpellier, Montpellier, France

[3]Department of Physics, Sungkyunkwan University, Suwon 16419, Republic of Korea

[4]Integrated Metabolomics Research Group, Metropolitan Seoul Center, Korea Basic Science Institute, Seoul 03759, Republic of Korea

[5]College of Pharmacy, Chung-Ang University, Seoul 06974, Republic of Korea

[6]Biomedical Institute for Convergence at SKKU and [7]Department of Metabiohealth, Sungkyunkwan University, Suwon 16419, Republic of Korea

[†]These authors contributed equally: Yeongmok Lee and Elsa Demes-Causse

[*]Corresponding author: sangholee@skku.edu

Supplementary Figures 1 – 21

Supplementary Tables 1 – 3

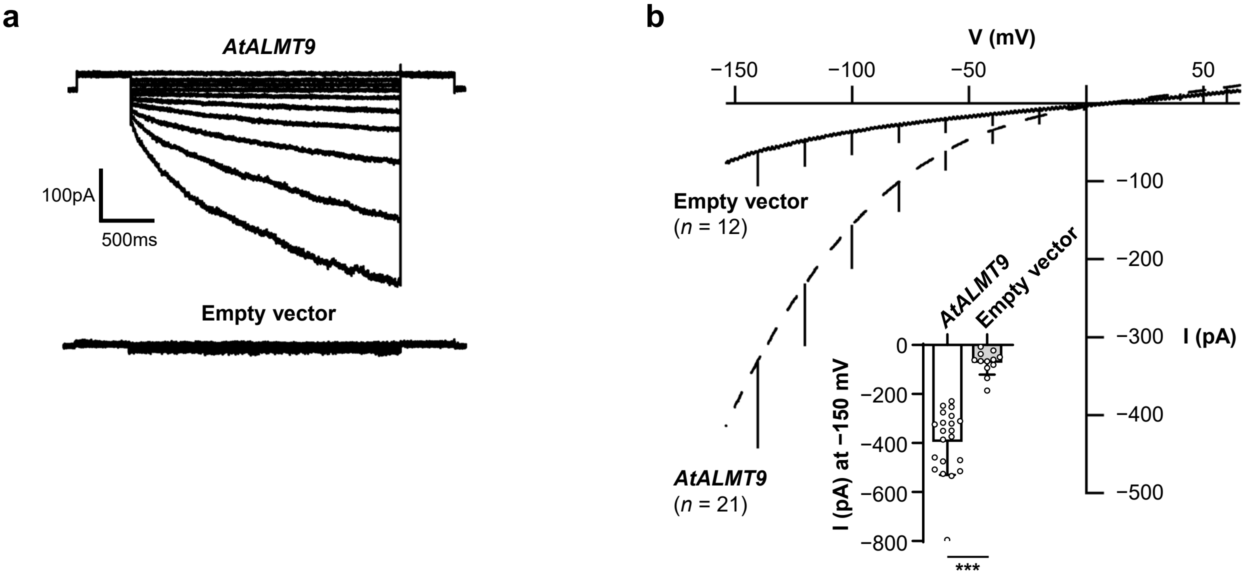

Supplementary Figure 1. Vacuolar patches over-expressing *AtALMT9* in *N. benthamiana*.
a, Representative currents from excised cytosolic-side-out vacuolar patches overexpressing ALMT9 and empty-vector-transformed in *N. benthamiana*. After a pre-pulse at +60 mV, voltage were applied for 2.5 s from +66 mV to −114 mV in −20 mV decrements, holding potential was 0 mV. **b,** Mean current-voltage characteristics from vacuolar patches overexpressing *AtALMT9* and empty-vector-transformed in *N. benthamiana*. Currents were evoked with a 3 s ramp from +66 to −154 mV. *AtALMT9* ($n = 21$; dashed line), empty vector ($n = 12$; full line). Error bars represent the standard deviation. Inset, Mean current intensity at −150 mV. Each data was represented as mean ± standard deviation with data points shown. Statistical analysis was done with non-parametric two-sided Mann-Whitney test. P value is < 0.000001. *$P < 0.05$; **$P < 0.01$; ***$P < 0.0001$; ns, not statistically significant.

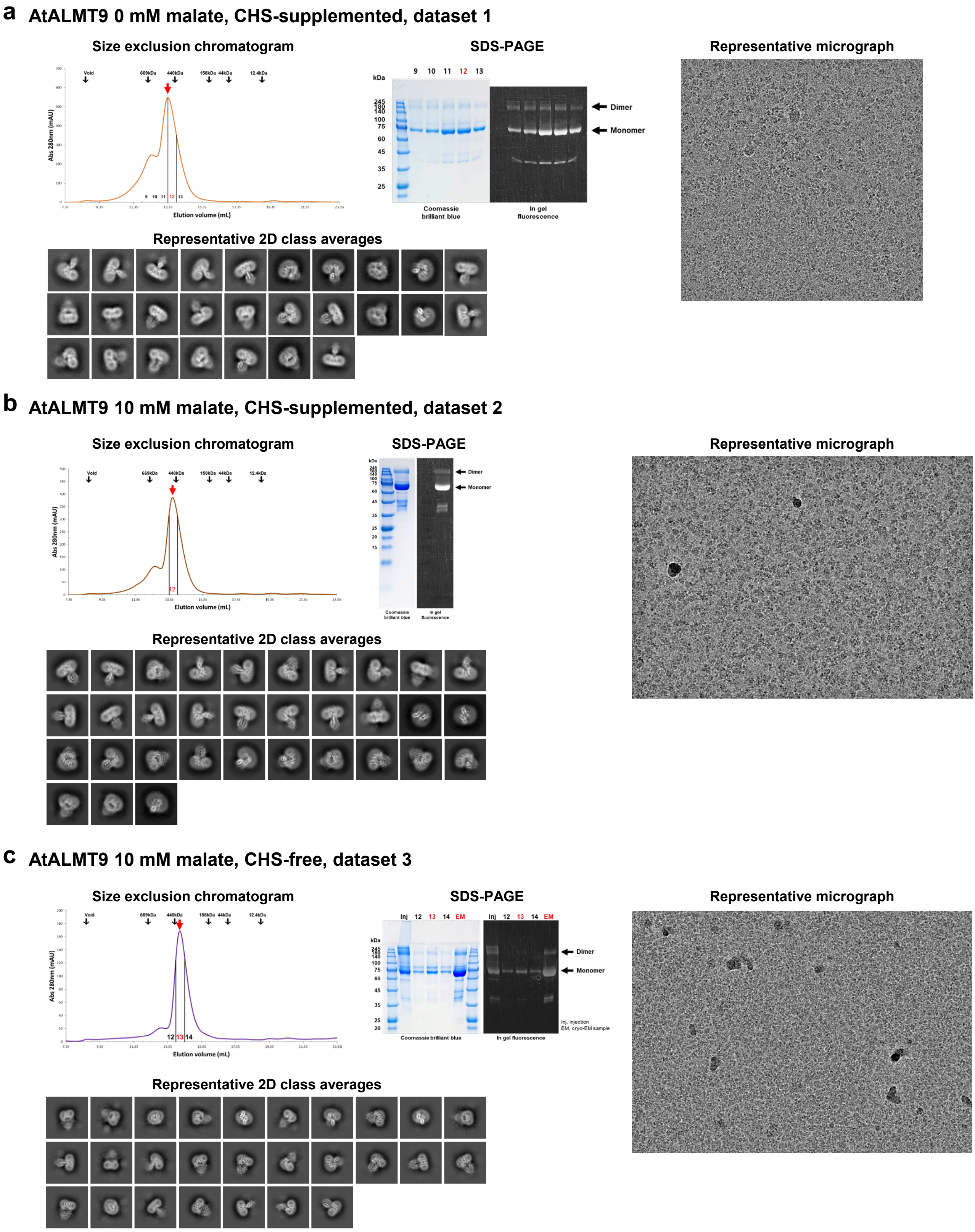

Supplementary Figure 2. Protein purifications and Cryo-EM analysis of AtALMT9-sfGFP-HA-H_{10} datasets.

a, Dataset 1 with LMNG and CHS. **b,** Dataset 2 with LMNG, CHS, and malate. **c,** Dataset 3 with LMNG and malate. A representative size exclusion chromatogram of each dataset is shown along with representative SDS-PAGE gels stained by Coomassie brilliant blue and detected by fluorescence. A peak of each dataset containing cryo-EM samples is indicated by a red arrows and fraction numbers. A representative micrograph of each dataset is shown. Representative 2D class averages of each dataset are shown.

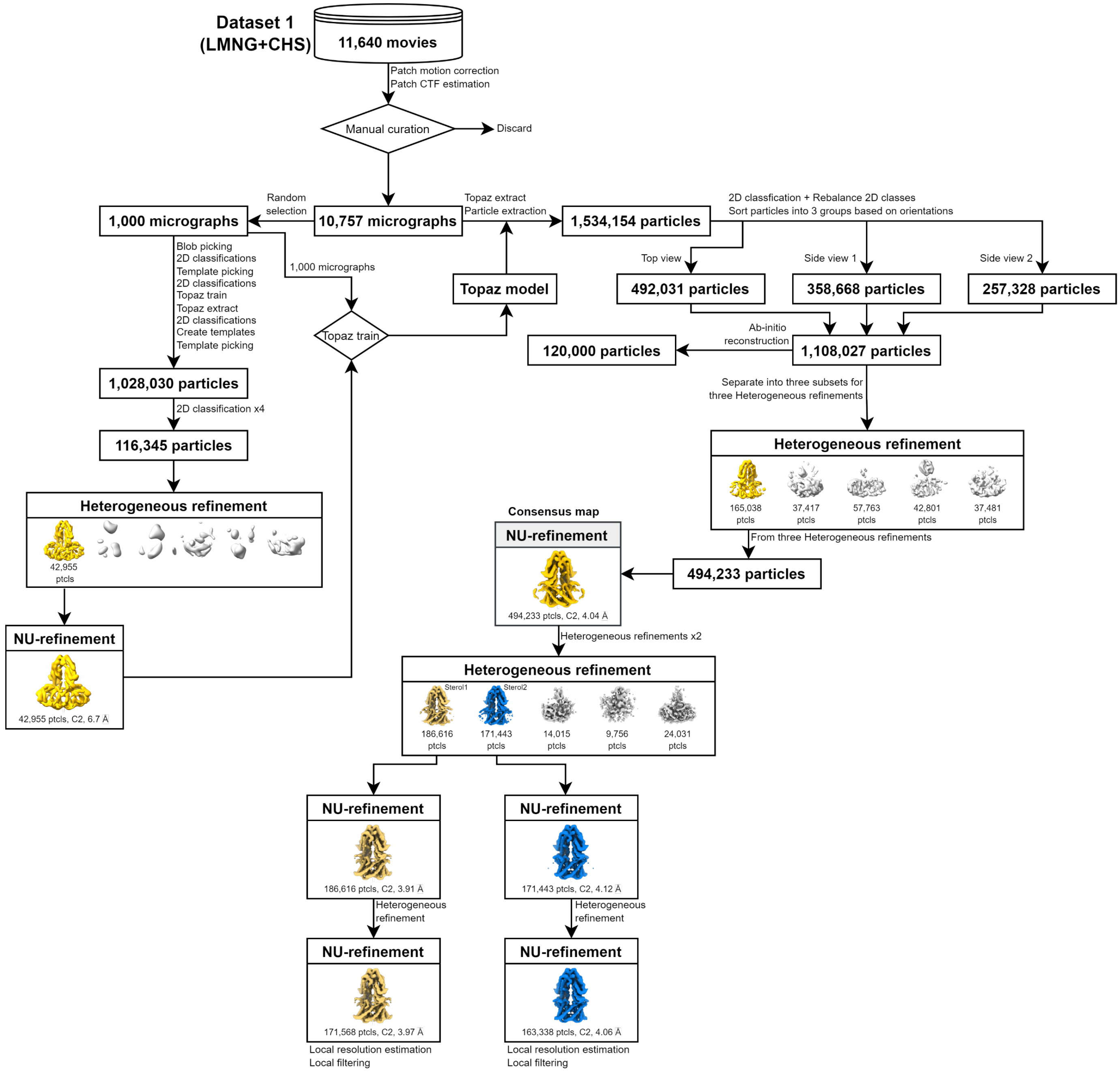

Supplementary Figure 3. Data processing workflow for AtALMT9 malate-free, CHS-supplemented dataset 1.
Cryo-EM data processing were performed using cryoSPARC[1]. See Methods for details.

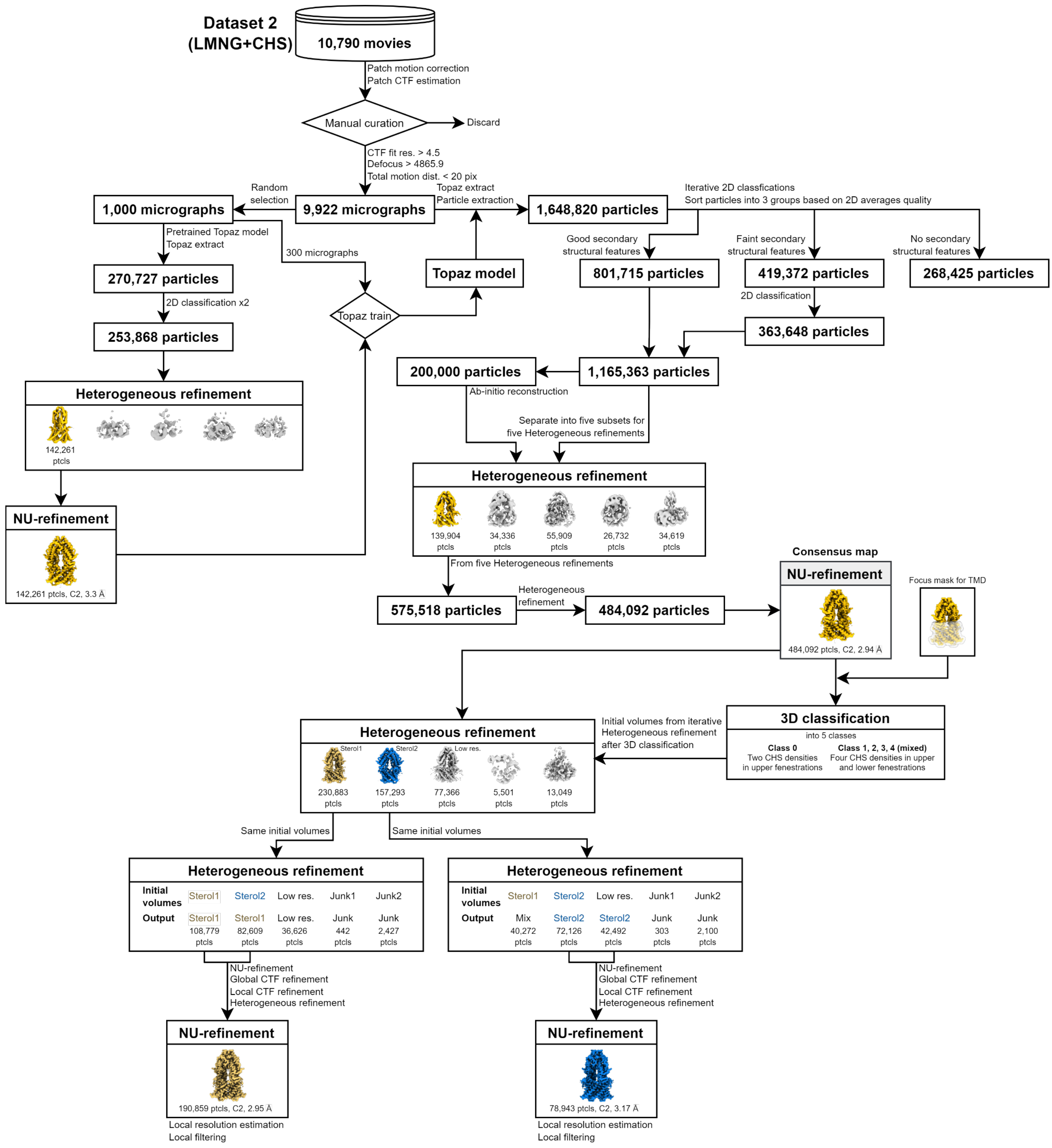

Supplementary Figure 4. Data processing workflow for AtALMT9 malate-added, CHS-supplemented dataset 2.

Cryo-EM data processing were performed using cryoSPARC[1]. See Methods for details.

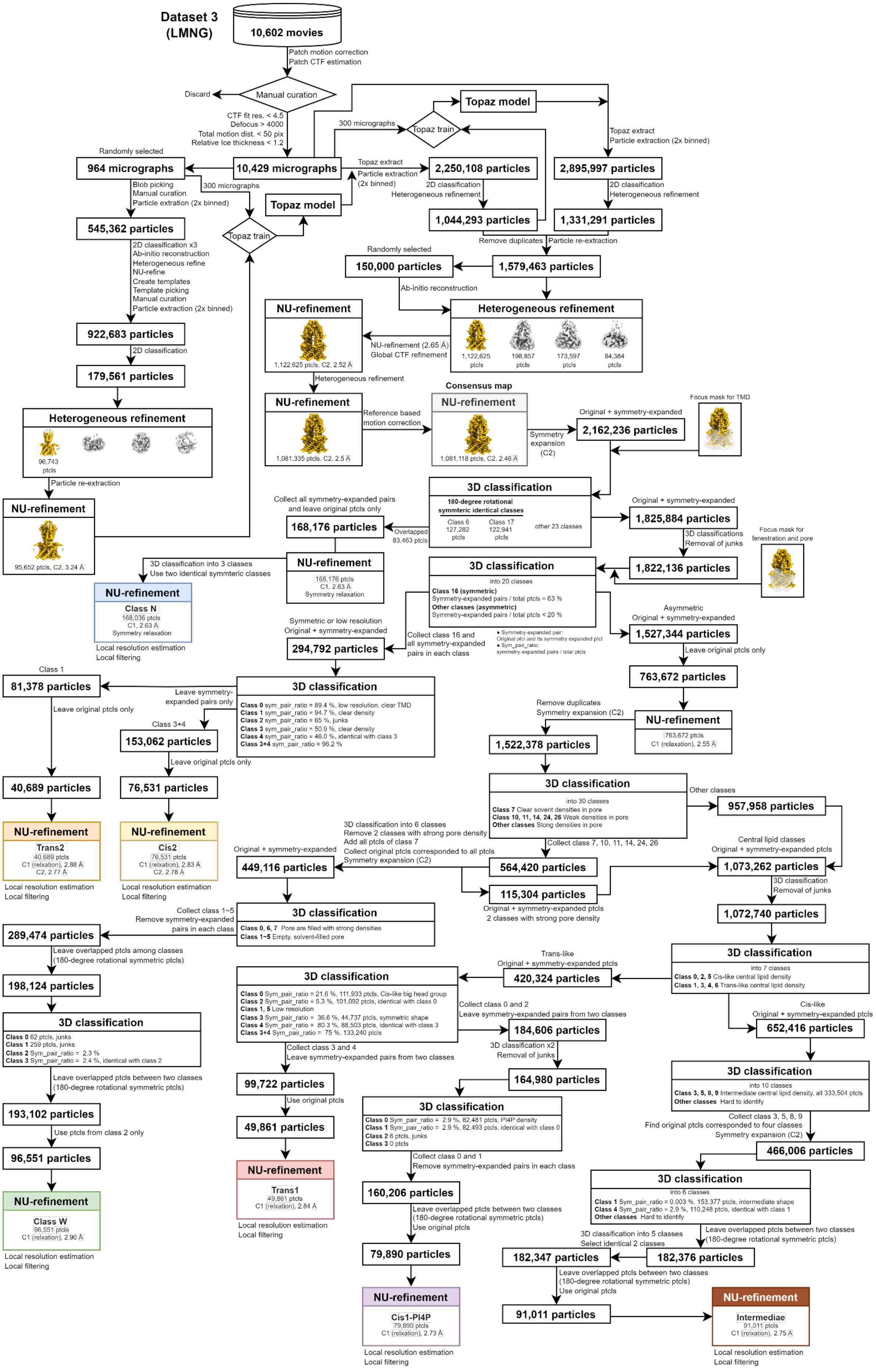

Supplementary Figure 5. Data processing workflow for AtALMT9 malate-added, CHS-free dataset 3. Cryo-EM data processing were performed using cryoSPARC[1]. See Methods for details.

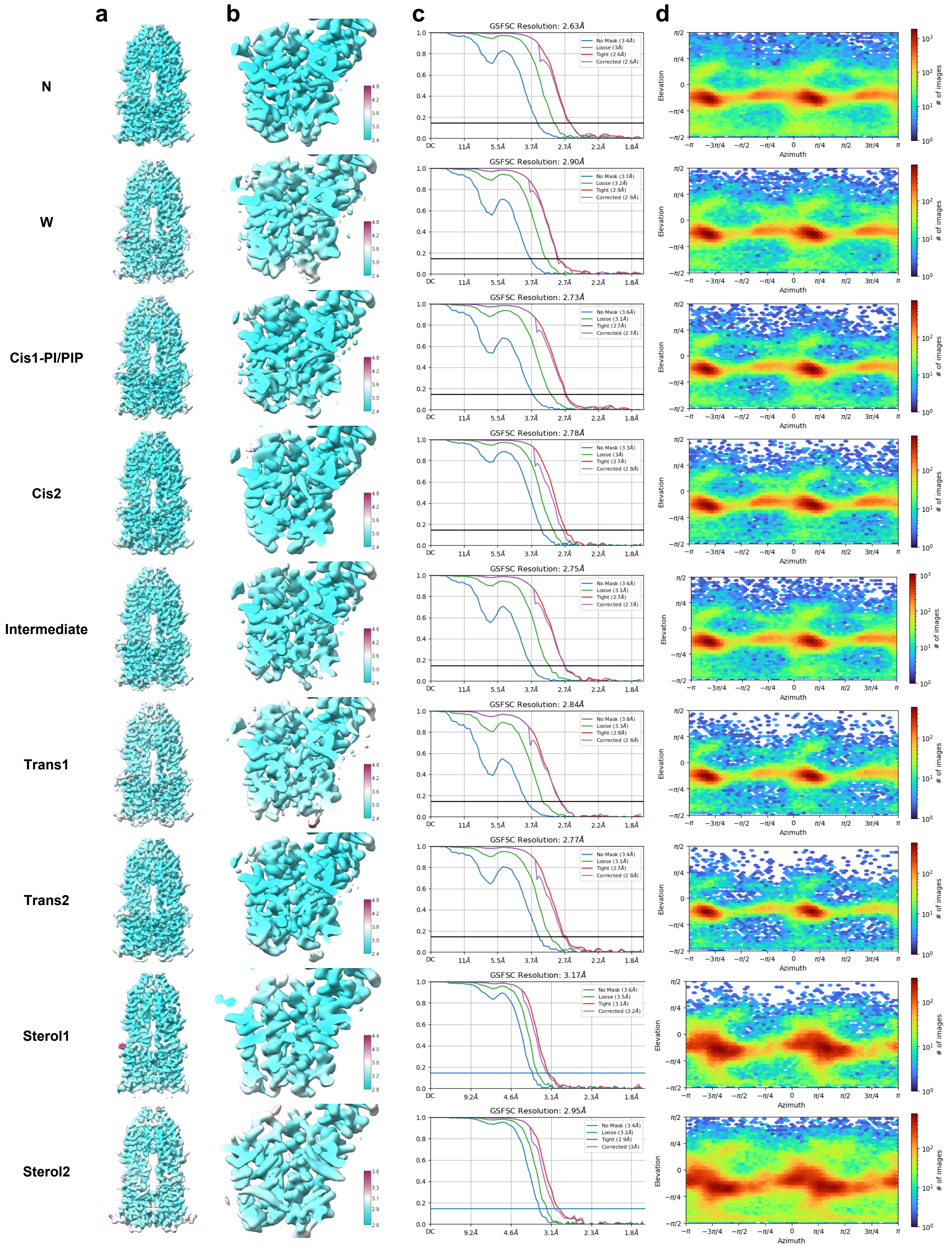

Supplementary Figure 6. Data quality of cryo-EM maps.

a, Local resolution estimations for unsharpened cryo-EM map. **b,** Local resolution estimations for pore and fenestration region. **c,** Golden-standard Fourier shell correlation (GSFSC) curves for cryo-EM map. **d,** Euler angle distributions.

a

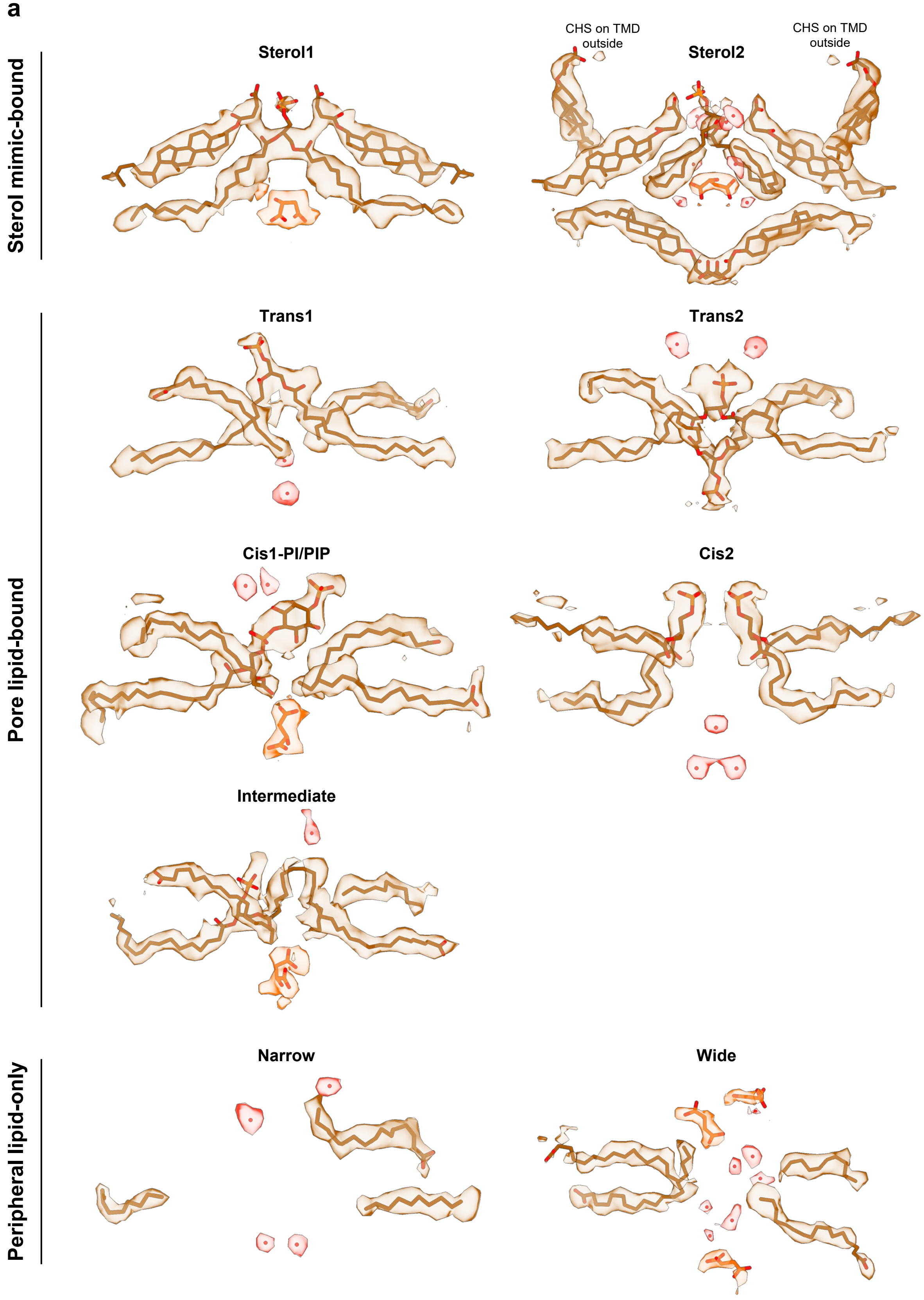
Sterol mimic-bound
Sterol1
Sterol2
CHS on TMD outside
CHS on TMD outside
Pore lipid-bound
Trans1
Trans2
Cis1-PI/PIP
Cis2
Intermediate
Peripheral lipid-only
Narrow
Wide

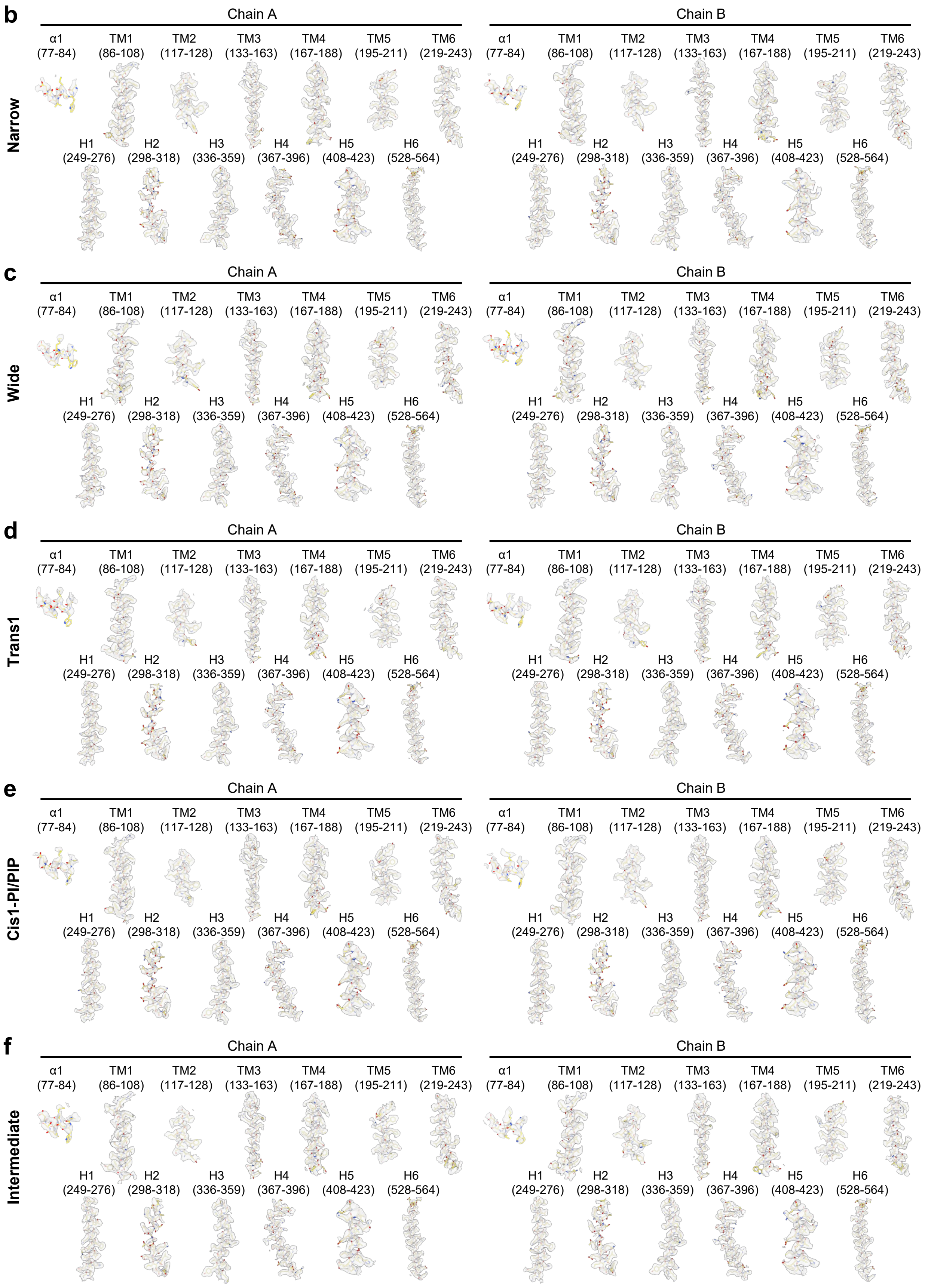

Supplementary Figure 7. Map densities and models of lipid, solvent molecules, and helices.
a, Map densities and models of lipids and solvent molecules of all states. Lipids and solvent molecules are depicted as stick representations. Lipids are colored as brown; malate as orange; water as red. Electron densities corresponding to lipid, water, and malate molecules, are sharpened and contoured at 6.7 σ for Narrow, Wide, and Trans1; 6 σ for Intermediate and Cis2; 5.5 σ for Trans2; 5 σ for Cis1-PI/PIP; 4 σ for Sterol1 and Sterol2 classes. **b-f,** Map densities and models of helices in Narrow, Wide, Trans1, Cis1-PI/PIP, and Intermediate classes. Residues are depicted as stick representations. Electron densities are sharpened and contoured at 6 σ for Wide; 5.5 σ for Narrow, Trans1, Cis1-PI/PIP, and Intermediate classes.

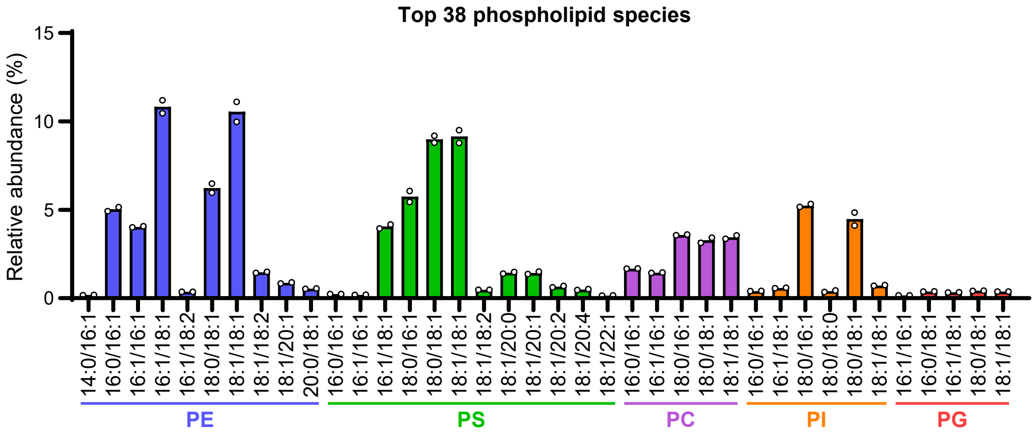

Supplementary Figure 8. Relative abundance of extracted phospholipids from purified AtALMT9. Relative abundance of top 38 phospholipid species detected from mass spectrometry. Each data was represented as mean with data points shown.

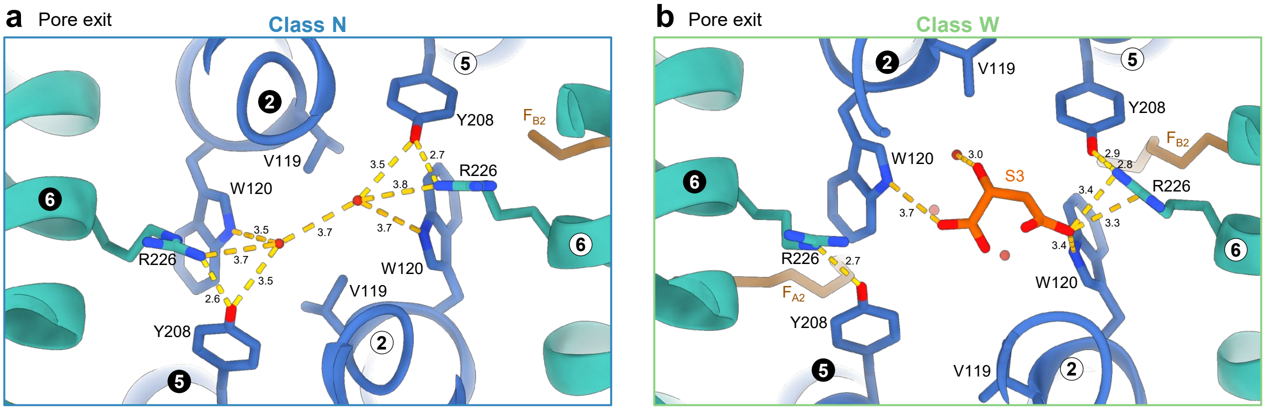

Supplementary Figure 9. Comparison of pore exit regions in the classes N and W.
Pore exit regions of the classes **a,** N (narrow) and **b,** W (wide). TMDs are depicted as cartoon with key residues in stick representations. Lipids, malate, and water molecules are depicted as stick representations. Pore helices are colored as blue; fenestration helices as teal; lipids as brown; water molecules as red; malate molecules as orange. The circled numbers in circles indicate transmembrane helices. The circle colors indicate protomer A (white number in black circle) and protomer B (black number in white circle). Distances between two atoms in Å are described along with dashed lines. Malate binding sites are labeled as “S3”.

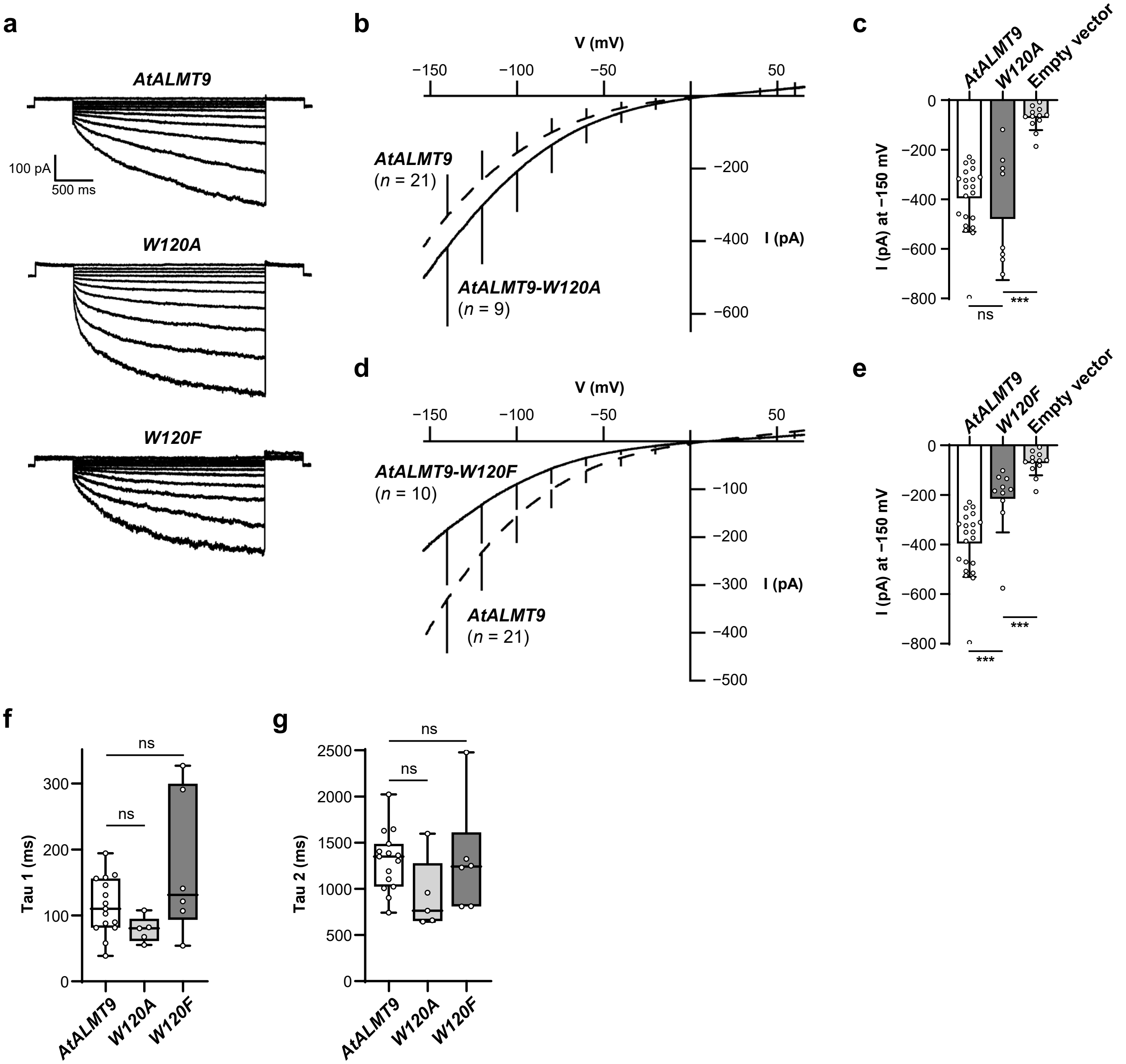

Supplementary Figure 10. Vacuolar patches over-expressing *AtALMT9*, *AtALMT9-W120A*, and *AtALMT9-W120F* in *N. benthamiana*.

a, Representative currents from vacuolar patches over-expressing *AtALMT9*, *AtALMT9-W120A*, and *AtALMT9-W120F* in *N. benthamiana*. After a pre-pulse at +60 mV, voltage steps were applied for 2.5 s from +66 mV to −114 mV in −20 mV decrements, holding potential was 0 mV. **b and d,** Mean current-voltage characteristics from vacuolar patches over-expressing *AtALMT9* (*n* = 21; dashed line; **b** and **d**) and *AtALMT9-W120A* (*n* = 9; full line; **b**) and *AtALMT9-W120F* (*n* = 10; full line; **d**). Currents evoked with a 3 s ramp from +66 mV to −154 mV. **c and e,** Mean current intensity at −150 mV. Each data was represented as mean ± standard deviation with data points shown. **f and g,** Box plots of the time constants Tau 1 (**f**) and Tau 2 (**g**) estimated from the curve fitting using a double exponential equation (see Methods) for *AtALMT9* (*n* = 15), *AtALMT9-W120A* (*n* = 5), and *AtALMT9-W120F* (*n* = 6). Each data was represented as a box for the mean and quartiles, whiskers for the minimum and maximum, and data points shown. Statistical analysis was done with non-parametric two-sided Mann-Whitney test. *P* values in (**c**) are 0.48 and 0.000027, respectively. *P* values in (**e**) are 0.00022 and 0.00030, respectively. *P* values in (**f**) are 0.066 and 0.13, respectively. *P* values in (**g**) are 0.053 and 0.52, respectively.; **P* < 0.05; ***P* < 0.01; ****P* < 0.0001; ns, not statistically significant.

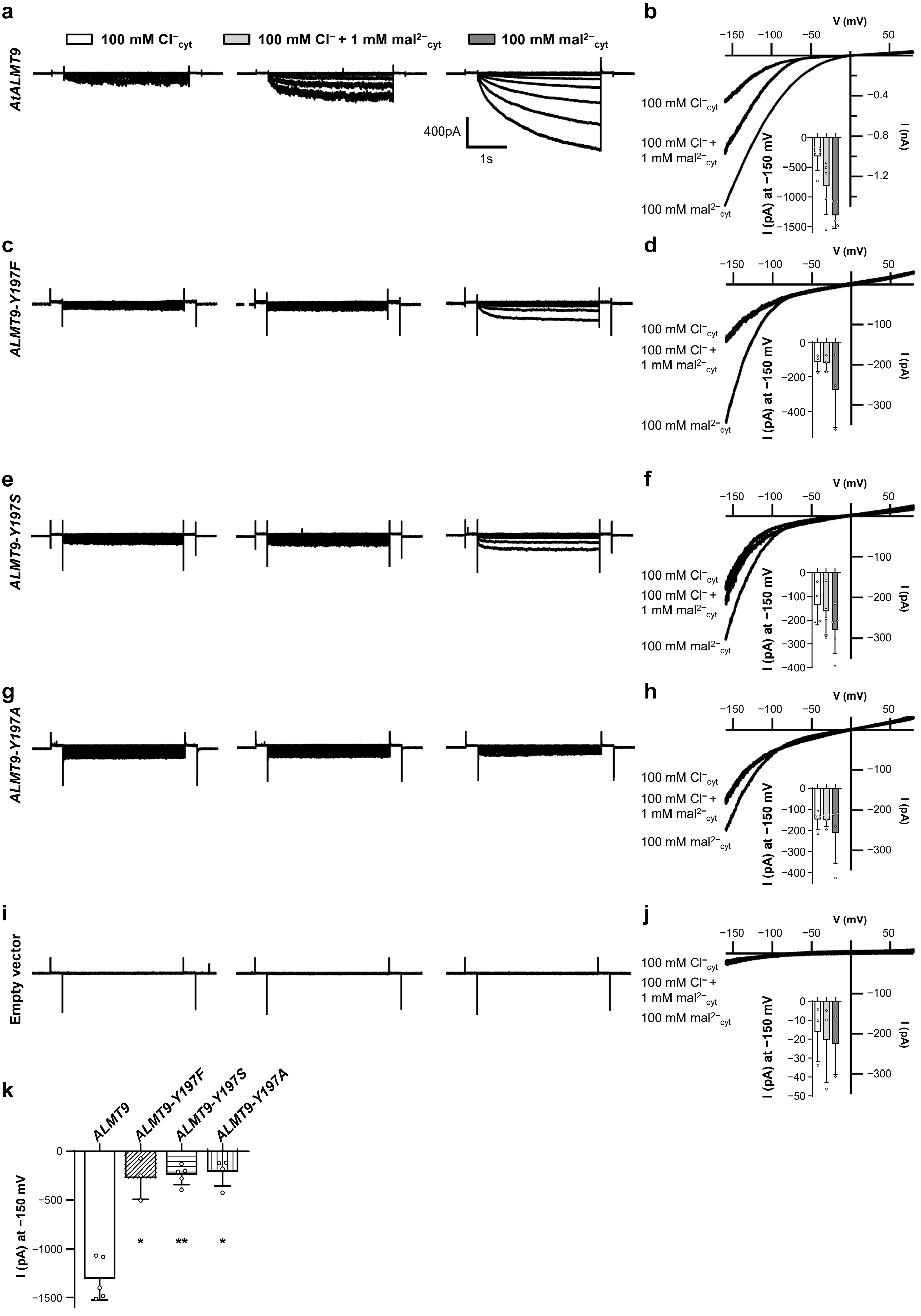

a
100 mM Cl⁻cyt
100 mM Cl⁻ + 1 mM mal²⁻cyt
100 mM mal²⁻cyt
AtALMT9
400pA
1s
b
V (mV)
−150 −100 −50 50
100 mM Cl⁻cyt
100 mM Cl⁻ + 1 mM mal²⁻cyt
100 mM mal²⁻cyt
I (pA) at −150 mV
I (nA)
c
ALMT9-Y197F
d
e
ALMT9-Y197S
f
g
ALMT9-Y197A
h
i
Empty vector
j
I (pA)
k
ALMT9
ALMT9-Y197F
ALMT9-Y197S
ALMT9-Y197A
I (pA) at −150 mV
*
**
*

Supplementary Figure 11. Effect of Y197 mutations on *AtALMT9* ion transport activity in different cytosolic conditions.

a, c, e, g, and i, Representative currents from excised vacuolar cytosolic-side-out over-expressing *AtALMT9* (**a**), *AtALMT9-Y197F* (**c**), *AtALMT9-Y197S* (**e**), *AtALMT9-Y197A* (**g**) and an empty vector (**i**) in *N. benthamiana.* Vacuolar patches were sequentially perfused with 100 mM $chloride_{cyt}$ (left); 100 mM chloride + 1 mM $malate_{cyt}$ (center); $malate_{cyt}$ in 100 mM $chloride_{vac}$ (right) buffers. After a prepulse at +80 mV, currents were evoked by a 3s pulse starting at +80 until −120 mV with −20 mV decrement, a holding potential at 0 mV. **b, d, f, h, and j,** Mean current-voltage characteristics from excised cytosolic-side-out vacuolar patches evoked with a 3 s voltage ramps starting from +80 to −160 mV. Insets, Histograms representing the current averages measured at −150 mV in the three tested cytosolic conditions (100 mM $chloride_{cyt}$ white; 100 mM chloride + 1 mM $malate_{cyt}$ light grey; $malate_{cyt}$ dark grey) for *AtALMT9* (**b**; $n = 5$), *AtALMT9-Y197F* (**d**; $n = 3$), *AtALMT9-Y197S* (**f**; $n = 5$), *AtALMT9-Y197A* (**h**; $n = 4$) and an empty vector (**j**; $n = 3$). Each data was represented as mean ± standard deviation with data points shown. **k,** Mean current intensities of *AtALMT9* ($n = 5$), *AtALMT9-Y197F* ($n = 3$), *AtALMT9-Y197S* ($n = 5$), *AtALMT9-Y197A* ($n = 4$) at −150 mV. Each data was represented as mean ± standard deviation with data points shown. Statistical analysis was done with non-parametric two-sided Mann-Whitney test. *P* values are 0.036, 0.0079, and 0.016, respectively. $*P < 0.05$; $**P < 0.01$; $***P < 0.0001$; ns, not statistically significant. Currents were recorded in the following ion conditions: *cytosolic side*: 1) 100 mM malic acid, 0.1 mM $CaCl_2$, pH 7.5 with Bis-Tris Propane, osmolarity 500 mOsmol with sorbitol; 2) 100 mM HCl, 1 mM malic acid, 0.1 mM $CaCl_2$, pH 7.5 with Bis-Tris Propane, osmolarity 500 mOsmol with sorbitol; 3) 100 mM HCl, 0.1 mM $CaCl_2$, pH 7.5 with Bis-Tris Propane, osmolarity 500 mOsmol with sorbitol; *vacuolar side*: 11.2 mM malic acid; 100 mM HCl; pH 6 with Bis-Tris Propane and osmolarity 550 mOsmol with sorbitol.

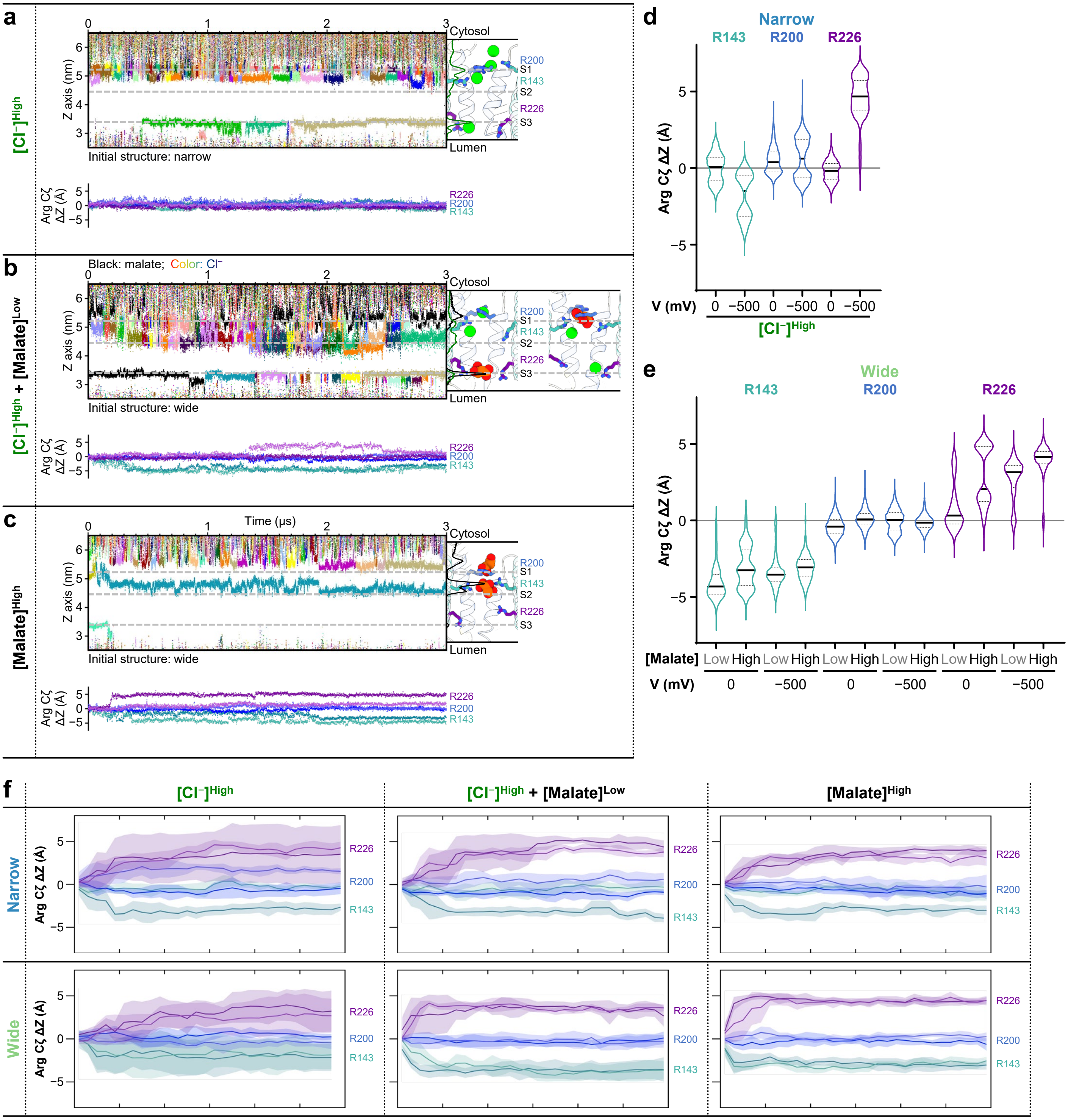

Supplementary Figure 12. Arginine movements driven by membrane potential.

a-c, Anion trajectory plots along the pore (Z axis) of AtALMT9 TMD (77-248) in MD simulations at zero membrane potential are shown. The unplugged classes and anion compositions used in each simulation are labeled. Representative distributions of anions and configuration of key arginine residues in the pore are depicted in the right-hand side of each panel: cartoons for protein; line graphs for anion distributions along the Z axis; sticks for key arginine residues R143, R200, and R226; green spheres for chloride; red and orange spheres for malate. Grey dashed lines indicate the anion binding sites, labeled as "S1", "S2", and "S3". The bottom side graphs indicate positional changes of key arginine residues (Cζ) relative to initial positions. **d-e,** Distribution plots of key arginine residues (Cζ) in simulations. Violin plots are used to visualize the positional distribution of Cζ atoms along the z-axis during simulations. Means and quartiles are indicated by thick solid and thin dashed lines, respectively. **f,** Mean positional changes of key arginine residues (Cζ) relative to initial positions throughout three independent simulations for 3 μs at −500 mV. Each data was represented as a thick line for the mean and a transparent area for the standard deviation.

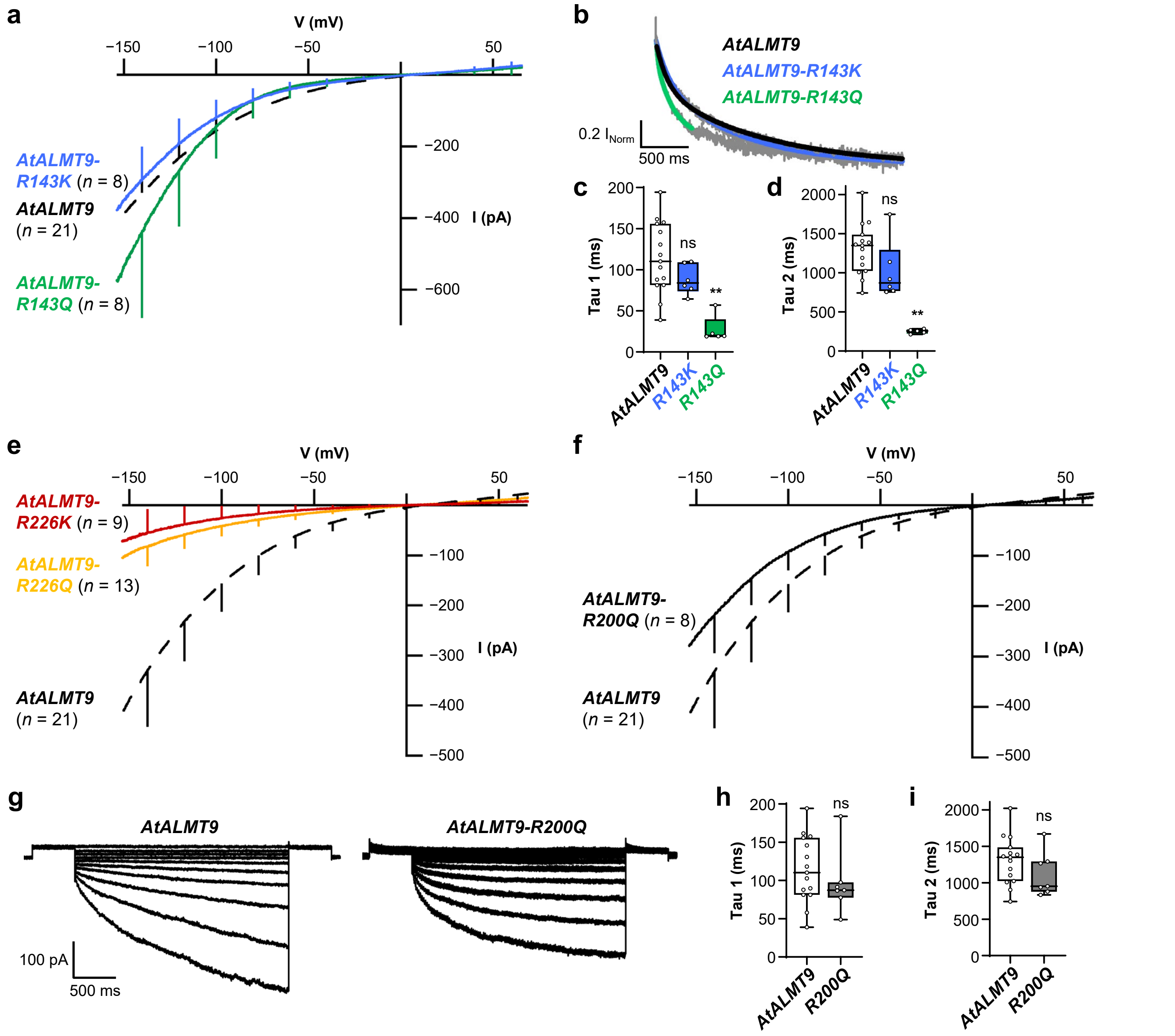

Supplementary Figure 13. Vacuolar patches over-expressing *AtALMT9, AtALMT9-R143K, AtALMT9-R143Q, and AtALMT9-R200Q* in *N. benthamiana*.

a, e, and f, Mean current-voltage characteristics from vacuolar patches over-expressing *AtALMT9* (*n* = 21; dashed line; **a**, **e**, and **f**), *AtALMT9-R143K* (*n* = 8; blue; **a**), *AtALMT9-R143Q* (*n* = 8; green; **a**), *AtALMT9-R226K* (*n* = 9; red; **e**), *AtALMT9-R226Q* (*n* = 13; yellow; **e**) and *AtALMT9-R200Q* (*n* = 8; black; **f**). Currents evoked with a 3 s ramp from +66 mV to −154 mV. **b**, Representative normalized current traces (light grey) fitted with a double exponential curve from vacuolar patches over-expressing *AtALMT9* (black), *AtALMT9-R143K* (blue), *AtALMT9-R143Q* (green). **c, d, h, and i,** Box plots of the time constants Tau1 and Tau2 estimated from the curve fitting using a double exponential equation (see material and methods) for *AtALMT9* (*n* = 15; white; **c**, **d**, **h**, and **i**) *AtALMT9-R143K* (*n* = 6; blue; **c** and **d**), *AtALMT9-R143Q* (*n* = 5; green; **c** and **d**) *AtALMT9-R200Q* (*n* = 7; dark grey; **h** and **i**). Each data was represented as a box for the mean and quartiles, whiskers for the minimum and maximum, and data points shown. **g,** Representative currents from vacuolar patches over-expressing *AtALMT9* and *AtALMT9-R200Q* in *N. benthamiana*. Statistical analysis was done with non-parametric two-sided Mann-Whitney test. *P* values in (**c**) are 0.11 and 0.00026, respectively. *P* values in (**d**) are 0.11 and 0.00013, respectively. *P* value in (**h**) is 0.30. *P* value in (**i**) is 0.16. *$P < 0.05$; **$P < 0.01$; ***$P < 0.0001$; ns, not statistically significant.

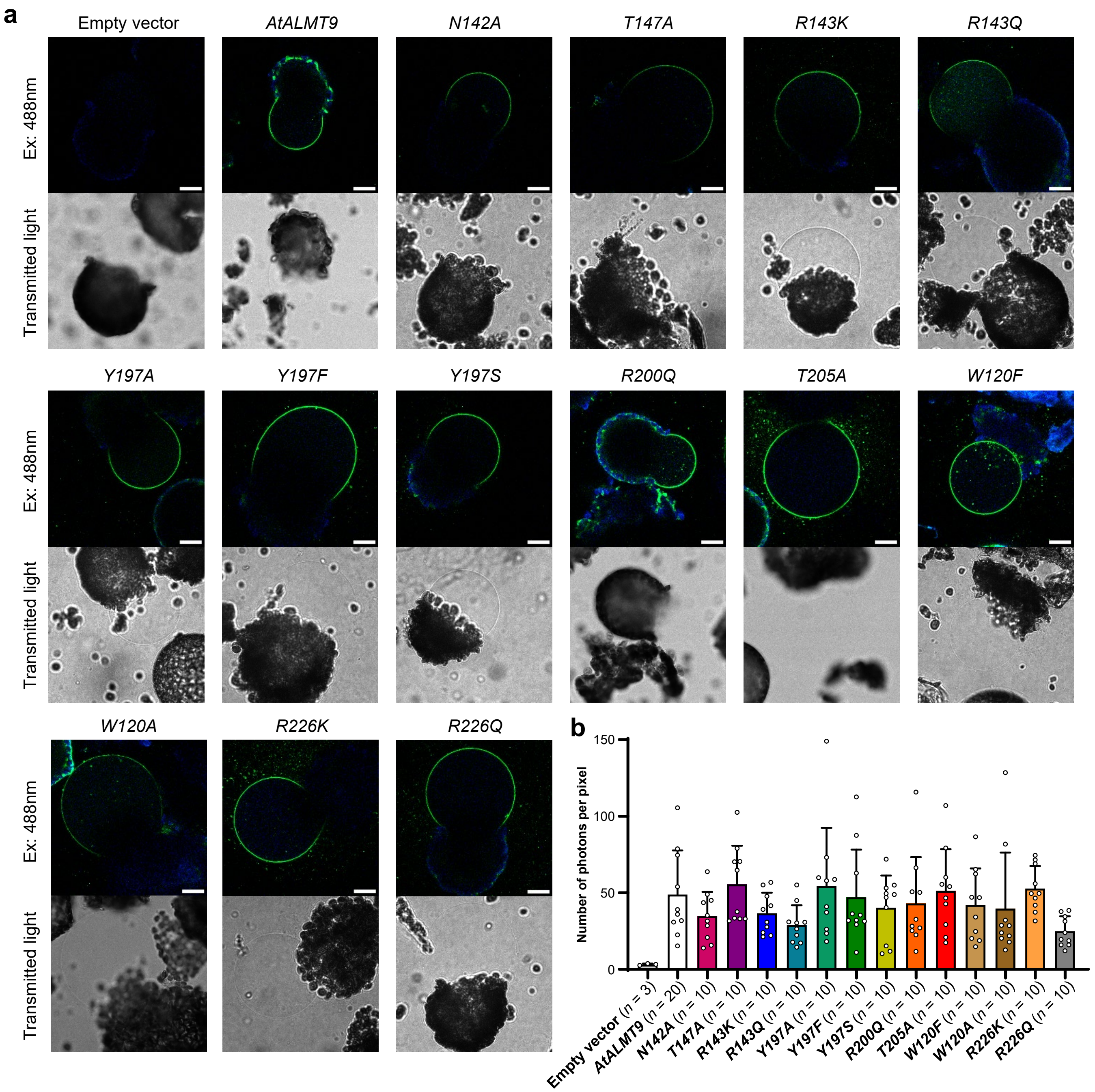

Supplementary Figure 14. Confocal images of *N. benthamiana mesophyll* vacuoles over-expressing AtALMT9 and mutants.

a, Confocal images of a vacuole released from a protoplast isolated after agroinfiltration of *N. benthamiana* mesophyll of an empty vector, AtALMT9 and all tested mutants with a GFP tag. GFP fluorescence was detected after excitation at 488 nm and emission at 500–550 nm and 600–650 nm (GFP: green; Chlorophyll: blue; top) and corresponding transmitted light images (bottom). Scale bars: 20 µm. **b,** Photon quantification at the vacuolar membrane for each tested construct. Each data was represented as mean ± standard deviation with data points shown. Number of observations are indicated in the parenthesis.

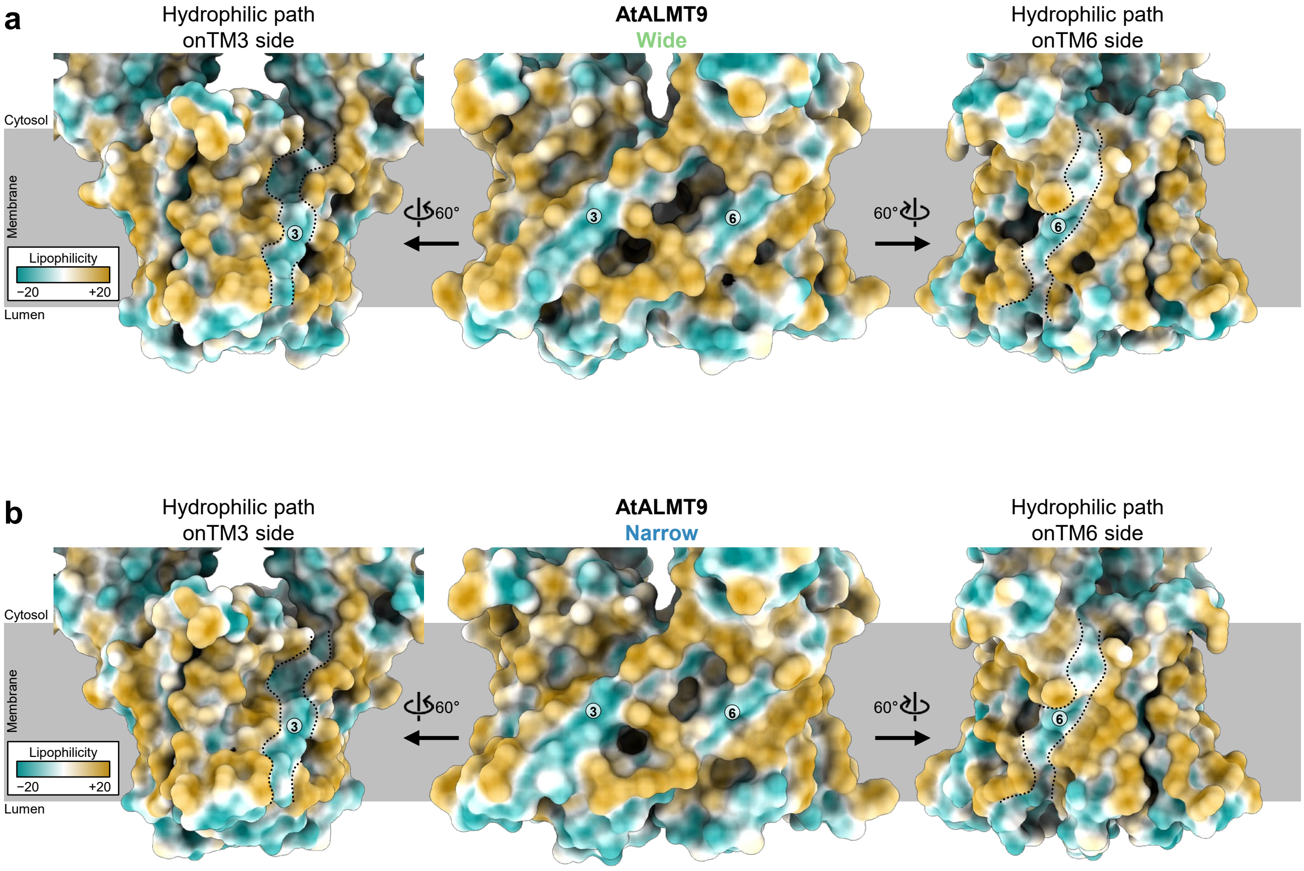

Supplementary Figure 15. Comparison of fenestration regions in the classes W and N. Lipophilicity around fenestration region of the AtALMT9 classes W (**a**) and N (**b**). Surfaces are colored with indicated color according to lipophilicity. Membrane is depicted as gray box. The numbers in circles indicate transmembrane helices. Center is the front fenestration view. left and right are the 60-degree rotated front views as indicated directions.

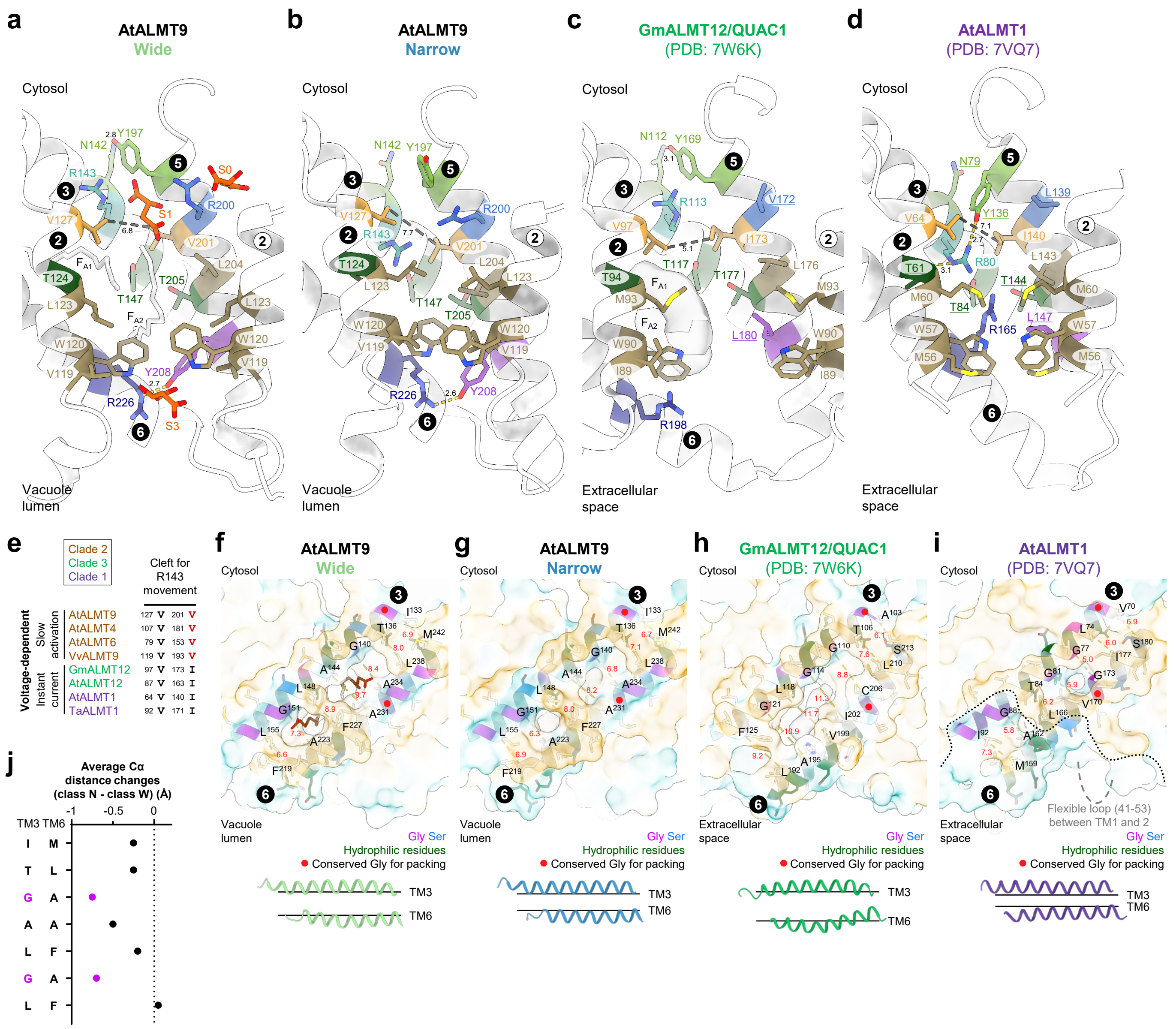

Supplementary Figure 16. Key residue comparison of ALMT structures.

a-d, Comparison of pore regions of the AtALMT9 class W (**a**) and N (**b**) structures, open state GmALMT12/QUAC1 structure (7W6K) (**c**), and open state AtALMT1 structure (7VQ7) (**d**). The pore regions are depicted as cartoon representation of the same view as in Fig. 2e and f. Functionally important residues, lipid and malate in the pore are depicted as sticks. Residues are color based on their roles in AtALMT9. Corresponding residues of GmALMT12 and AtALMT1 are colored as same color. Underlined residues indicate significant differences in their roles compared to those of AtALMT9. **e,** Cleft residues from multiple sequence alignment results of various ALMTs with known voltage-dependent kinetics. **f-i,** Fenestration view of wide (**f**) and narrow (**g**) state of AtALMT9, open state GmALMT12/QUAC1 structure (7W6K) (**h**) and open state AtALMT1 structure (7VQ7) (**i**). Fenestration is depicted as surface. Fenestration helices are depicted as cartoon and stick representations. The open state structures are aligned against transmembrane domain of one protomer of AtALMT9 wide class. Distances between TM3 and 6 are indicated by distances between Cα atoms of two residues in bold. Interhelical residues are labeled. TM3 and 6 are depicted as thin cartoon representations to show the curvature. **j,** Cα distance changes of interhelical residues from the N to W class. Interhelical distances from two protomers are used to obtain average distances.

Description on Supplementary Figure 16

Among the three pore arginine residues of AtALMT9 (R143, R200, and R226), R143 and R226 are strictly conserved. By contrast, R200 is substituted with aliphatic residues in GmALMT12 (V172) and in AtALMT1 (L139) (**a-d**). Residues corresponding to R200 are arginine in clade 2 with voltage-dependent slow activation, but not in other ALMTs with voltage-dependent instantaneous current (Fig. 5d). Despite the strict conservation of R143 and R226 (Fig. 5d), their rotamers and mobilities appear different (**a-d**). For instance, AtALMT9 has up- and down-pointed R143 and down-pointed R226 in the basal states (**a, b**). GmALMT12 shows R113 (R143 of AtALMT9) pointing upward and R198 (R226 of AtALMT9) downward (**c**), whereas AtALMT1 R80 (R143 of AtALMT9) downward and R165 (R226 of AtALMT9) upward, which is similar to the conductive state of AtALMT9 in the MD simulations (**d**).

The cleft residues underneath R143 and R200 are largely conserved but with some variations. Unlike the wide cleft between V127 and V201 (6.8 Å for class W and 7.7 Å for class N) in AtALMT9, that of V97 and I173 (5.1 Å) in GmALMT12 is too narrow for downward movement of R113 (**a-c, e**). AtALMT1 has a similarly wide cleft between V64 and I140 (7.1 Å), but Y136 rotates downward through wide cleft and fastens R80 (R143 of AtALMT9) downward by hydrogen bonding (**d, e**).

The pore lock residues show differential conservation levels. The cytosolic lock residues N142 and Y197 are strictly conserved. Y197 of AtALMT9 class W and Y169 of GmALMT12 interact with N142 and N112, respectively. By contrast, such pairing is not observed between Y136 and N79 of AtALMT1 (**a, c**). The interaction between Y197 and N142 in AtALMT9 apparently contributes to stabilizing the wide pore states (**a, b**). Consistent with this structural observation, their mutation dramatically reduces AtALMT9 functionality (Fig. 2i-k, Supplementary Fig. 11). The pair residues Y208 and R226 forming the basal lock is not conserved. Y208 interacts with R226 to fix its down-position in all basal states, except the sterol2 class (**a, b**). The residues corresponding to Y208 in other ALMTs (L180 of GmALMT12 and L147 of AtALMT1) are not conserved, but act as part of the hydrophobic lining of the pore (**c, d**). The pore constriction residues are not well conserved, except residues corresponding to W120 and L204 of AtALMT9 (**a-d**).

Two fenestration helices in the W class of AtALMT9 and in GmALMT12 are convexly curved, whereas those of the N class of AtALMT9 and AtALMT1 are almost flat (**f-i**). Glycine and proline residues function as helix breakers, thereby introducing a helical kink or curvature[2,3]. While no proline residue is found, 9 out of 15 glycine residues in TMD are located in the fenestration helices of AtALMT9. However, there is no connection between number of glycine residues (6 out of 15 residues of GmALMT12/QUAC1 TMD; 10 out of 16 residues of AtALMT1 TMD) and curvature of the fenestration helices in ALMT structures (**f-i**). In AtALMT1, the glycine residues are also correlated with tight packing of TM3 and 6 (**i**). The one glycine-glycine pair shows the closest contact (5.0 Å) and the two inter-helical residue pairs containing one glycine residue show closer contacts than others (**i**). We measured the Cα distances of the interhelical residues between TM3 and 6 to elucidate side chain effects on fenestration shrinking from the wide to narrow class (**j**). Glycine also contributes to packing of transmembrane helices due to absence of its side chain[4,5]. The two glycine-alanine pairs of TM3 and 6 exhibit the largest shrinking, and one alanine-alanine pair also represents a larger change (**j**). By contrast, aromatic and large aliphatic residues show small average distance changes in two protomers (**j**).

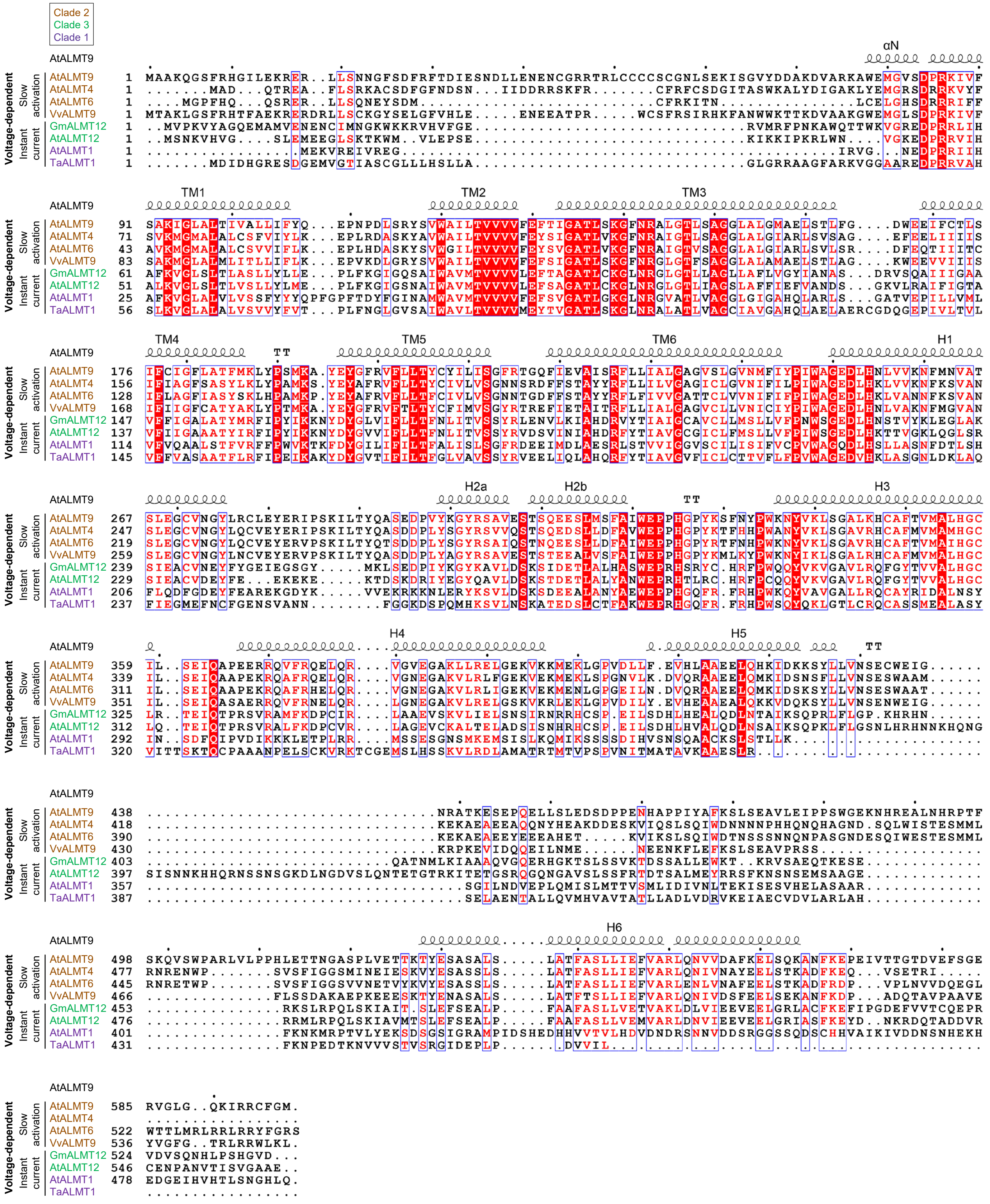

Supplementary Figure 17. Multiple sequence alignment of ALMT clades 1, 2 and 3.

Multiple sequence alignment of ALMT clade 1, 2 and 3: AtALMT9 in clade 2; its paralogous AtALMT4 and 6; its orthologous VvALMT9; AtALMT12 in clade 3; its orthologous GmALMT12; AtALMT1 in clade 1; its orthologous TaALMT1. The sequence alignment was performed by ClustalX[6] and illustrated by ESPript3[7].

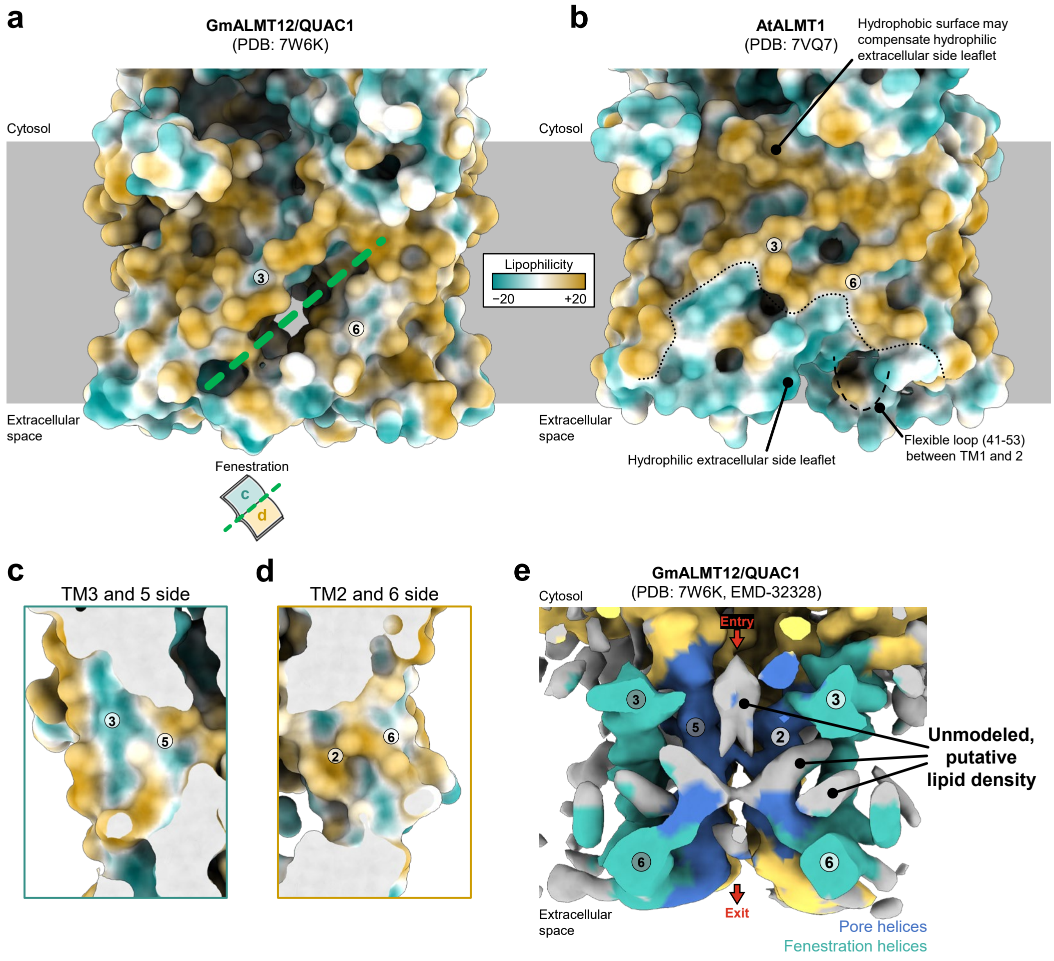

Supplementary Figure 18. Fenestration regions of other ALMT structures.

a-b, Lipophilicity around fenestration region of GmALMT12/QUAC1 (PDB: 7W6K) and AtALMT1 (PDB: 7VQ7, pH 5, Al^{3+}) structures. Surfaces are colored with indicated color according to lipophilicity. Membrane is depicted as gray box. The numbers in circles indicate helices. Green dashed lines, teal and yellow pages of open book indicate direction of slice and view in (**c**) and (**d**). **c-d,** Slice views of fenestration of GmALMT12/QUAC1 (PDB: 7W6K). **e,** Slice views of pore and fenestration regions of GmALMT12/QUAC1 (PDB: 7W6K) map (EMD-32328). Electron densities are unsharpened and contoured at 5.5 σ. Pore helices are colored as blue, fenestration helices as teal, other modeled regions as yellow, unmodelled regions as gray.

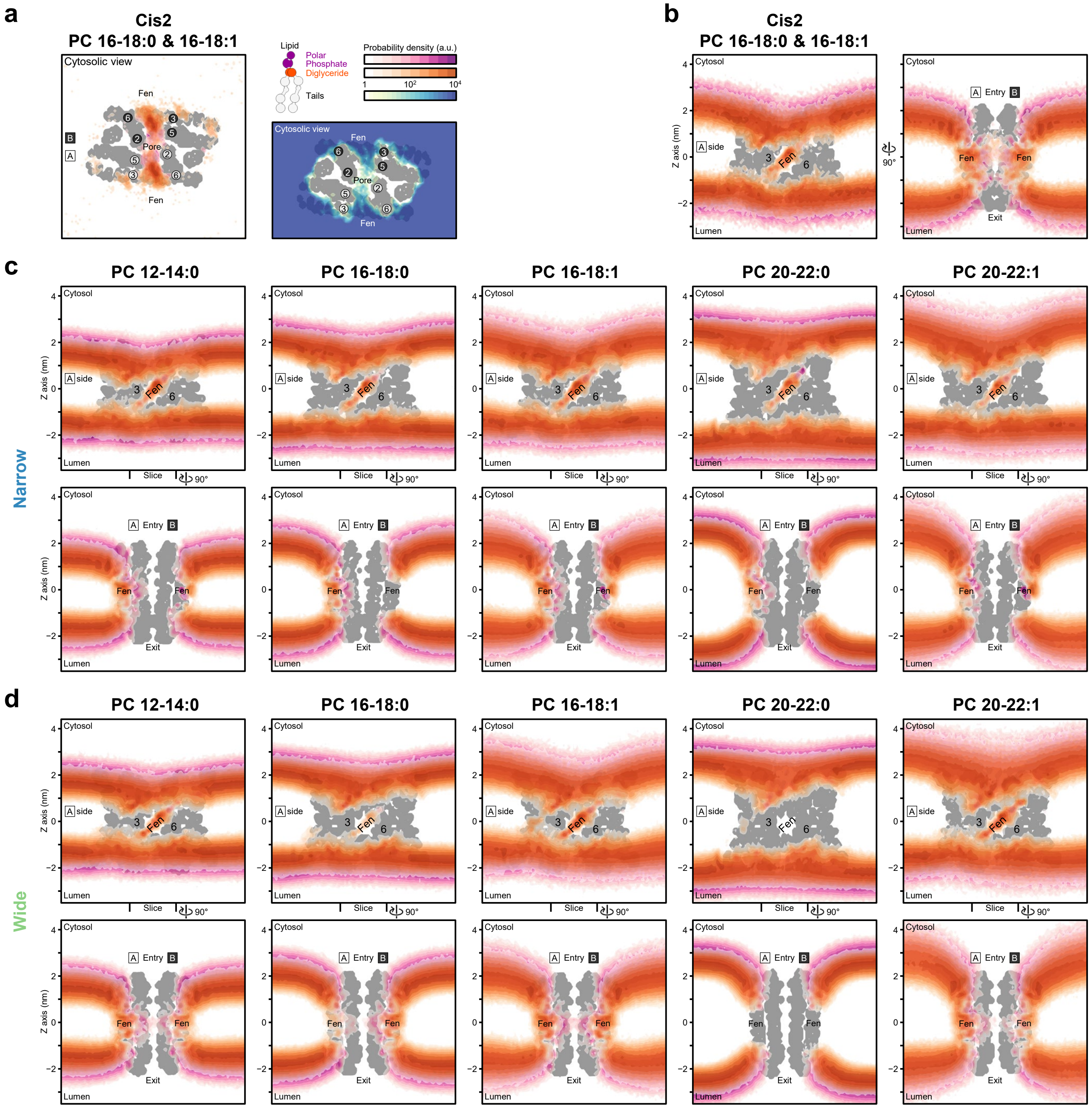

Supplementary Figure 19. Lipid migration through hydrophilic fenestration regions.
Probability distributions for the lipid moieties in the coarse-grained simulations. Probability of each lipid moiety is depicted using the indicated color gradient (**a**, right) in ascending order: white to purple for polar and phosphate; white to orange for diglyceride; and yellow to blue for tails. **a,** Clipped cytosolic views for the hydrophilic lipid moieties (left) and the hydrophobic lipid tails (right) in the cis2 class. **b-d,** Clipped side views for the hydrophilic lipid moieties in the cis2 class (**b**), the narrow (N) class (**c**), and the wide (W) class (**d**). The AtALMT9 TMD structure classes and membrane lipids used in simulations are labeled. The initial lipids in the cis2 class simulation are modeled as phosphatidylethanolamine (PE) with symmetric 16-18:1 tails for pore lipids; phosphatidylcholine (PC) with asymmetric 16-18:0 and 16-18:1 tails for membrane lipids. The membrane lipids in the N and W classes are PC with various symmetric tails and only one tail is labeled. The circle or box colors indicate protomer A (white number in black background) and protomer B (black number in white background). The numbers indicate transmembrane helices. The positions of the cytosol, lumen, pore, entry, exit, and fenestrations (fen) are labeled.

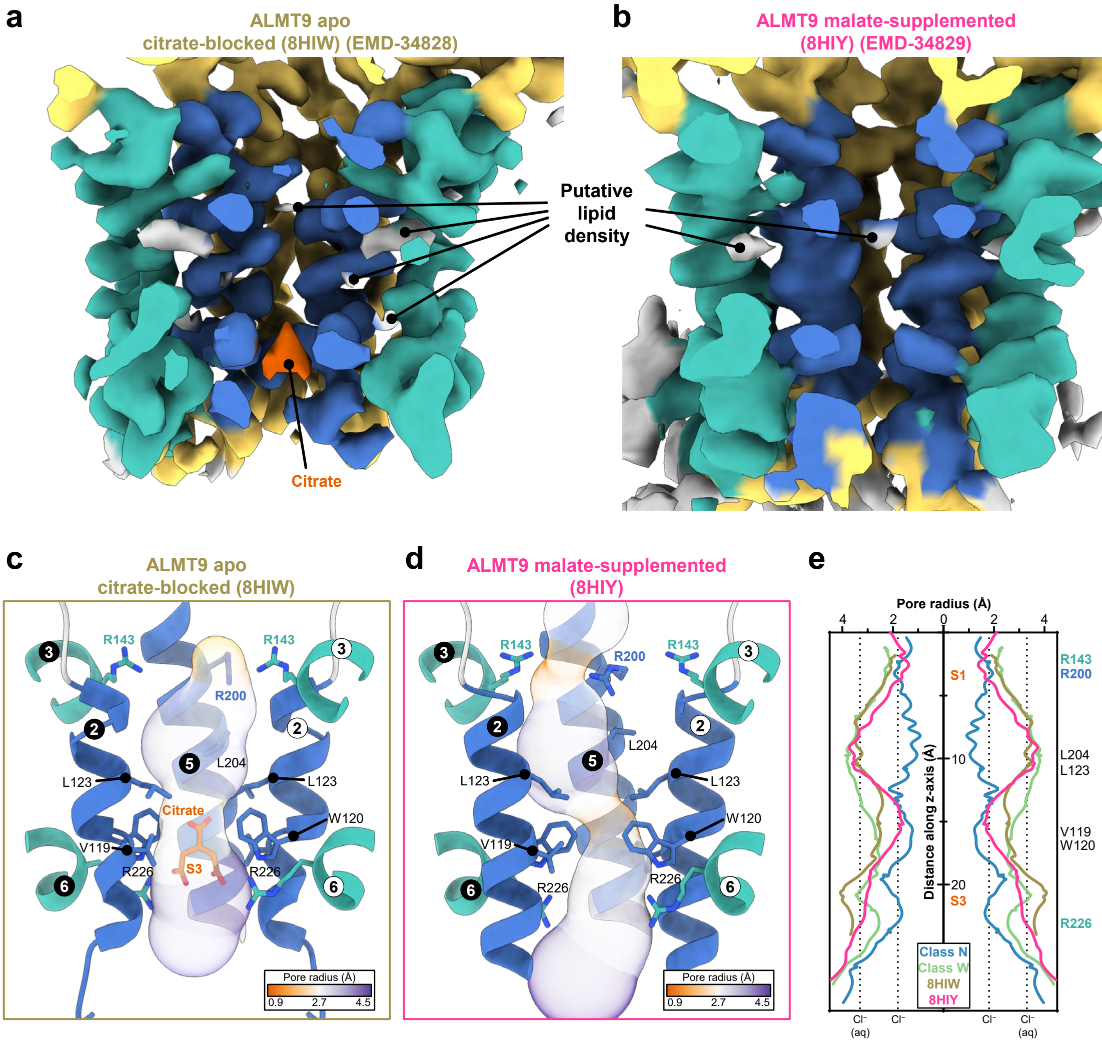

Supplementary Figure 20. Conductance pathway in other ALMT9 structures.
a-b, Central map slices of recently reported ALMT9 structures in citrate-blocked (PDB: 8HIW) and malate-supplemented (PDB: 8HIY) states. Pore helices are colored as blue, fenestration helices as teal, other modeled regions as yellow, unmodelled regions as gray. **c-d,** Conductance pathways of ALMT9 structures. TMDs are depicted as cartoon with key residues in stick representations. Pores are depicted as transparent surface and colored with indicated color according to pore radii. Pore helices are colored as blue, fenestration helices as teal, and citrate molecule as orange. The numbers in circles indicate helices. The circle colors indicate protomer A (white number in black circle) and protomer B (black number in white circle). Citrate binding sites are labeled as "S3". **e,** Pore radius plot of class O, C, citrate-blocked (PDB: 8HIW) and malate-supplemented (PDB: 8HIY) structures. Pore-lining residues are labeled on corresponding position. Radii for ionic (Cl^-) and hydrated chloride ions (Cl^- (aq)) are indicated by vertical dot lines. Malate binding sites in pore, "S1" and "S3", are labeled on corresponding position.

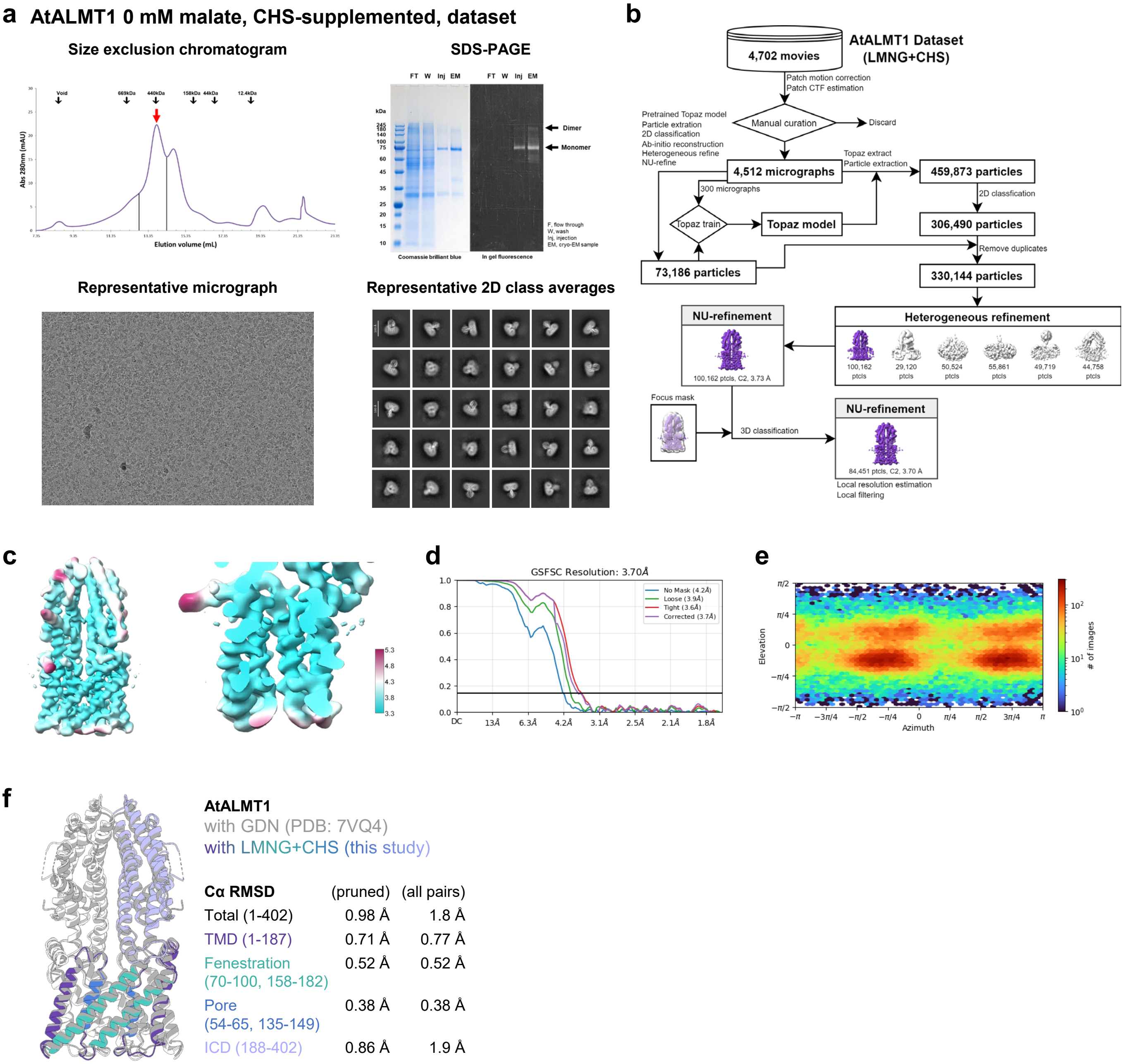

Supplementary Figure 21. Cryo-EM analysis of AtALMT1-sfGFP-HA-H_{10} dataset

a, Protein purification and cryo-EM analysis results of AtALMT1-sfGFP-HA-H_{10} CHS-supplemented dataset. Representative size exclusion chromatogram. Representative SDS-PAGE gel stained by Coomassie brilliant blue and detected by fluorescence to check purity of sample. Peak containing cryo-EM sample is indicated by a red arrow and label. Representative micrograph. Representative 2D class averages. **b,** Cryo-EM data processing scheme of AtALMT1 dataset using cryoSPARC[1]. See Methods for details. **c,** Local resolution estimations for unsharpened cryo-EM map. The right panel is slice view for pore and fenestration region. **d,** Golden-standard Fourier shell correlation (GSFSC) curves for cryo-EM map. **e,** Euler angle distributions. **f,** Superposition of AtALMT1 structures in GDN or LMNG+CHS micelles. Structures are depicted as cartoon representation. Pore helices are colored as blue, fenestration helices as teal, TMD remnant as Purple, ICD as light purple, protomer B as white, and the structure with GDN micelle as gray. Cα RMSDs of each region are described in right panel.

Supplementary Table 1. Cryo-EM data collection, refinement and validation statistics

	**AtALMT9 Dataset 2 (LMNG+CHS)**		**AtALMT9 Dataset 3 (LMNG)**							**AtALMT1 (LMNG +CHS)**
	**Sterol1**<br>EMDB-60459<br>PDB 8ZTE	**Sterol2**<br>EMDB-60461<br>PDB 8ZTG	**N**<br>EMDB-60462<br>PDB 8ZTH	**W**<br>EMDB-60463<br>PDB 8ZTI	**Cis1-PI/PIP**<br>EMDB-60464<br>PDB 8ZTJ	**Cis2**<br>EMDB-60465<br>PDB 8ZTK	**Intermediate**<br>EMDB-60466<br>PDB 8ZTL	**Trans1**<br>EMDB-60467<br>PDB 8ZTM	**Trans2**<br>EMDB-60468<br>PDB 8ZTN	**Basal**<br>EMDB-60818<br>PDB 9JTW
**Data collection and processing**										
Magnification	105,000 x	105,000 x	105,000 x	105,000 x	105,000 x	105,000 x	105,000 x	105,000 x	105,000 x	100,000 x
Voltage (kV)	300	300	300	300	300	300	300	300	300	200
Electron exposure ($e^{-}/Å^2$)	60	60	50	50	50	50	50	50	50	50
Defocus range (μm)	−0.7 ~ −1.9	−0.7 ~ −1.9	−0.7 ~ −1.9	−0.7 ~ −1.9	−0.7 ~ −1.9	−0.7 ~ −1.9	−0.7 ~ −1.9	−0.7 ~ −1.9	−0.7 ~ −1.9	−0.7 ~ −1.9
Pixel size (Å)	0.85	0.85	0.858	0.858	0.858	0.858	0.858	0.858	0.858	0.84
Symmetry imposed	C2	C2	C1	C1	C1	C2	C1	C1	C2	C2
Initial particle images (no.)	1,648,820	1,648,820	2,895,997	2,895,997	2,895,997	2,895,997	2,895,997	2,895,997	2,895,997	459,873
Final particle images (no.)	78,943	190,859	168,036	96,551	79,890	76,531	91,011	49,681	40,689	84,451
Map resolution (Å)	3.17	2.95	2.63	2.90	2.73	2.78	2.75	2.84	2.77	3.70
FSC threshold	0.143	0.143	0.143	0.143	0.143	0.143	0.143	0.143	0.143	0.143
Map resolution range (Å)	2.8 ~ 12.0	2.7 ~ 10.3	2.5 ~ 38.7	2.5 ~ 43.2	2.5 ~ 42.3	2.5 ~ 32.2	2.5 ~ 43.9	2.6 ~ 45.5	2.5 ~ 39.7	3.4 ~ 14.6
**Refinement**										
Initial model used (PDB code)	Sterol2	AF-Q9LS46-F1 model v2	Sterol1	Trans2	Trans2	Trans2	Trans2	Trans2	Sterol1	7VQ4
Model resolution (Å)	3.2	3.0	2.8	3.0	2.8	2.8	2.9	3.0	2.8	3.8
FSC threshold	0.5	0.5	0.5	0.5	0.5	0.5	0.5	0.5	0.5	0.5
Map sharpening *B* factor ($Å^2$)	142.8	147.4	97.1	103.1	86.4	112.8	91.3	84.8	97.7	164.5
Model composition										
Non-hydrogen atoms	6360	6650	6167	6287	6326	6287	6311	6390	6304	6230
Protein residues	770	760	770	770	770	770	770	768	770	792
Ligands	4	8	3	7	4	2	5	3	2	-
B factors ($Å^2$)										
Protein	47.11	40.27	42.98	43.03	45.20	39.87	43.95	67.13	44.52	72.24
Ligand	37.42	28.59	33.68	44.83	37.73	40.92	40.64	53.88	37.11	-
R.m.s. deviations										
Bond lengths (Å)	0.003	0.005	0.003	0.002	0.002	0.002	0.003	0.003	0.002	0.002
Bond angles (°)	0.510	0.988	0.580	0.467	0.438	0.488	0.516	0.517	0.551	0.603
Validation										
MolProbity score	1.98	1.86	1.69	1.95	1.50	1.77	1.56	1.53	1.65	1.50
Clashscore	16.93	17.21	11.90	17.36	9.48	11.62	11.04	10.35	12.17	5.01
Poor rotamers (%)	0.94	0.31	0.78	0.94	0.31	0.62	0.00	0.47	0.78	0.00
Ramachandran plot										
Favored (%)	96.31	97.33	97.49	96.7	98.15	96.83	98.55	99.07	97.76	96.43
Allowed (%)	3.69	2.67	2.51	3.3	1.85	3.17	1.45	0.93	2.24	3.57
Disallowed (%)	0	0	0	0	0	0	0	0	0	0

Supplementary Table 2. Summary of all-atom simulation systems

System No.	Model	POPC	Na^+	Cl^-	$Malate^{2-}$	Water	Simulation time (μs)	Voltage (mV)
**1**	Narrow	156	31	31	0	11745	3.0	0
**2**	Wide	158	38	32	3	12002	3.0	0
**3**	Wide	158	86	0	43	11861	3.0	0
**4**	Narrow	156	41	41	0	11709	3.0	500
**5**	Narrow	156	80	0	40	11619	3.0	500
**6**	Narrow	156	47	41	3	11699	3.0	500
**7**	Wide	158	43	43	0	11979	3.0	500
**8**	Wide	158	86	0	43	11861	3.0	500
**9**	Wide	158	49	43	3	11979	3.0	500

Supplementary Table 3. Summary of coarse-grained simulation systems

System No.	Model	Lipid	# of Lipid	Na^+	Cl^-	Water	Simulation time (μs)	Remarks
**1**	Narrow	DLPC	178	42	42	3467	94.0	
**2**	Narrow	DOPC	164	39	39	3162	95.0	
**3**	Narrow	DPPC	182	32	32	2603	94.6	
**4**	Narrow	DBPC	206	52	52	4422	74.9	
**5**	Narrow	DGPC	188	42	42	3280	91.6	
**6**	Wide	DLPC	203	41	41	3320	94.8	
**7**	Wide	DOPC	187	44	44	3606	90.0	
**8**	Wide	DPPC	207	43	43	3598	100.1	
**9**	Wide	DBPC	196	42	42	3188	83.8	
**10**	Wide	DGPC	190	42	42	3390	77.9	
**11**	Cis2	POPC	190	43	43	3545	15.5	Two DOPE molecules inserted into the pore.

Supplementary References

1. Punjani, A., Rubinstein, J. L., Fleet, D. J. & Brubaker, M. A. cryoSPARC: algorithms for rapid unsupervised cryo-EM structure determination. *Nat. Methods* **14**, 290-296 (2017). https://doi.org:10.1038/nmeth.4169

2. Bright, J. N. & Sansom, M. S. P. The Flexing/Twirling Helix: Exploring the Flexibility about Molecular Hinges Formed by Proline and Glycine Motifs in Transmembrane Helices. *J. Phys. Chem. B* **107**, 627-636 (2003). https://doi.org:10.1021/jp026686u

3. Hogel, P. et al. Glycine Perturbs Local and Global Conformational Flexibility of a Transmembrane Helix. *Biochemistry* **57**, 1326-1337 (2018). https://doi.org:10.1021/acs.biochem.7b01197

4. Javadpour, M. M., Eilers, M., Eilers, M., Groesbeek, M. & Smith, S. O. Helix packing in polytopic membrane proteins: role of glycine in transmembrane helix association. *Biophys. J.* **77(3)**, 1609-1618 (1999). https://doi.org/10.1016/S0006-3495(99)77009-8

5. Kim, S. et al. Transmembrane glycine zippers: physiological and pathological roles in membrane proteins. *Proc. Natl. Acad. Sci. U. S. A.* **102**, 14278-14283 (2005). https://doi.org:10.1073/pnas.0501234102

6. Larkin, M. A. *et al.* Clustal W and Clustal X version 2.0. *Bioinformatics* **23**, 2947-2948 (2007). https://doi.org:10.1093/bioinformatics/btm404

7. Robert, X. & Gouet, P. Deciphering key features in protein structures with the new ENDscript server. *Nucleic Acids Res.* **42**, W320-W324 (2014). https://doi.org:10.1093/nar/gku316
