# Supplementary material for: Structural basis for malate-driven, pore lipid-regulated activation of the Arabidopsis vacuolar anion channel ALMT9
Source: Nat Commun. 2025 Feb 20;16:1817. doi: 10.1038/s41467-025-56940-5 (PMC11842843; doi:10.1038/s41467-025-56940-5)
Supplement: Supplementary file 2 — Description of Additional Supplementary Files [file 41467_2025_56940_MOESM2_ESM.pdf]

Description of Additional Supplementary Files

File Name: Supplementary Movie 1
Description: All-atom simulation of the narrow class with 200 mM chloride ions at 0 mV.

File Name: Supplementary Movie 2
Description: All-atom simulation of the wide class with 200 mM chloride ions and three malate molecules at 0 mV.

File Name: Supplementary Movie 3
Description: All-atom simulation of the wide class with 200 mM malate at 0 mV.

File Name: Supplementary Movie 4
Description: All-atom simulation of the narrow class with 200 mM chloride ions at −500 mV.

File Name: Supplementary Movie 5
Description: All-atom simulation of the wide class with 200 mM chloride ions at −500 mV.

File Name: Supplementary Movie 6
Description: All-atom simulation of the narrow class with 200 mM chloride ions and three malate molecules at −500 mV.

File Name: Supplementary Movie 7
Description: All-atom simulation of the wide class with 200 mM chloride ions and three malate molecules at −500 mV.

File Name: Supplementary Movie 8
Description: All-atom simulation of the narrow class with 200 mM malate at −500 mV.

File Name: Supplementary Movie 9
Description: All-atom simulation of the wide class with 200 mM malate at −500 mV.

File Name: Supplementary Movie 10
Description: Coarse-grained simulation of the narrow class in a DOPC membrane with 150 mM chloride ion at 0 mV.

File Name: Supplementary Movie 11
Description: Coarse-grained simulation of the wide class in a DOPC membrane with 150 mM chloride ion at 0 mV.

File Name: Supplementary Movie 12
Description: Coarse-grained simulation of the cis2 class in a POPC membrane with 150 mM chloride ion at 0 mV.

Descriptions on Supplementary Movies 1-12

All-atom or coarse-grained simulation movies of AtALMT9 TMD (77-248) for the indicated classes and conditions. Upper side is cytosolic side and lower side is luminal side. Representative anion distributions are depicted as surface; cartoon for protein; blue stick for R200; light blue stick for R143; violet stick for R226; brown stick for lipids; sphere for ions. One protomer is colored as white and the other as black. Chloride ions are colored as green; orange for malate molecules. In all-atom simulations at zero membrane potential, peripheral lipids and distributions within the fenestration are depicted using sphere and surface representations. In coarse-grained simulations, phosphate head groups are shown as red spheres.
