# Supplementary material for: Structural basis for malate-driven, pore lipid-regulated activation of the Arabidopsis vacuolar anion channel ALMT9
Source: Nat Commun. 2025 Feb 20;16:1817. doi: 10.1038/s41467-025-56940-5 (PMC11842843; doi:10.1038/s41467-025-56940-5)
Supplement: Supplementary file 15 — Reporting Summary [file 41467_2025_56940_MOESM15_ESM.pdf]

nature portfolio

Corresponding author(s): Sangho Lee

Last updated by author(s): Jan 15, 2025

Reporting Summary

Nature Portfolio wishes to improve the reproducibility of the work that we publish. This form provides structure for consistency and transparency in reporting. For further information on Nature Portfolio policies, see our Editorial Policies and the Editorial Policy Checklist.

Statistics

For all statistical analyses, confirm that the following items are present in the figure legend, table legend, main text, or Methods section.

n/a	Confirmed	
☐	☒	The exact sample size (*n*) for each experimental group/condition, given as a discrete number and unit of measurement
☐	☒	A statement on whether measurements were taken from distinct samples or whether the same sample was measured repeatedly
☐	☒	The statistical test(s) used AND whether they are one- or two-sided<br>*Only common tests should be described solely by name; describe more complex techniques in the Methods section.*
☒	☐	A description of all covariates tested
☒	☐	A description of any assumptions or corrections, such as tests of normality and adjustment for multiple comparisons
☐	☒	A full description of the statistical parameters including central tendency (e.g. means) or other basic estimates (e.g. regression coefficient) AND variation (e.g. standard deviation) or associated estimates of uncertainty (e.g. confidence intervals)
☐	☒	For null hypothesis testing, the test statistic (e.g. *F*, *t*, *r*) with confidence intervals, effect sizes, degrees of freedom and *P* value noted<br>*Give P values as exact values whenever suitable.*
☒	☐	For Bayesian analysis, information on the choice of priors and Markov chain Monte Carlo settings
☒	☐	For hierarchical and complex designs, identification of the appropriate level for tests and full reporting of outcomes
☒	☐	Estimates of effect sizes (e.g. Cohen's *d*, Pearson's *r*), indicating how they were calculated

Our web collection on statistics for biologists contains articles on many of the points above.

Software and code

Policy information about availability of computer code

Data collection	Cryo-EM data were collected using EPU software (Thermo Fisher Scientific)<br>Electrophysiological data were collected using PatchMaster software (HEKA)<br>Molecular dynamics system structures were prepared using CHARMM-GUI.<br>Parameters for the simulations were generated using CGenFF web server.<br>Molecular dynamics simulations were performed using GROMACS 2024.1.

Data analysis	Cryosparc v4.0 - 4.4<br>COOT v0.9.8.1<br>Phenix v1.20<br>UCSF ChimeraX v1.6.1<br>ClustalX v2.1<br>MOLE online (https://mole.upol.cz)<br>ESPript 3.0 (https://espript.ibcp.fr/ESPript/ESPript/)<br>GraphPad Prism 8.4.3<br>Heka Electronics (Fitmaster, 2x32, PatchMaster 2x30)<br>Fiji ImageJ (v1.54)<br>LasX (Leica)<br>CLC MainWorkBench (v20.0.4)<br>VMD 1.9.4a55

For manuscripts utilizing custom algorithms or software that are central to the research but not yet described in published literature, software must be made available to editors and reviewers. We strongly encourage code deposition in a community repository (e.g. GitHub). See the Nature Portfolio guidelines for submitting code & software for further information.

Data

Policy information about availability of data

All manuscripts must include a data availability statement. This statement should provide the following information, where applicable:

- Accession codes, unique identifiers, or web links for publicly available datasets
- A description of any restrictions on data availability
- For clinical datasets or third party data, please ensure that the statement adheres to our policy

Coordinates have been deposited in the Protein Data Bank under accession codes 8ZTE [http://doi.org/10.2210/pdb8zte/pdb] (AtALMT9 sterol1 class), 8ZTG [http://doi.org/10.2210/pdb8ztg/pdb] (AtALMT9 sterol2 class), 8ZTH [http://doi.org/10.2210/pdb8zth/pdb] (AtALMT9 N class), 8ZTI [http://doi.org/10.2210/pdb8zti/pdb] (AtALMT9 W class), 8ZTJ [http://doi.org/10.2210/pdb8ztj/pdb] (AtALMT9 cis1-PI/PIP class), 8ZTK [http://doi.org/10.2210/pdb8ztk/pdb] (AtALMT9 cis2 class), 8ZTL [http://doi.org/10.2210/pdb8ztl/pdb] (AtALMT9 intermediate class), 8ZTM [http://doi.org/10.2210/pdb8ztm/pdb] (AtALMT9 trans1 class), 8ZTN [http://doi.org/10.2210/pdb8ztn/pdb] (AtALMT9 trans2 class), and 9JTW [http://doi.org/10.2210/pdb9jtw/pdb] (AtALMT1). The corresponding cryo-EM density maps with local-filtering, half-maps and masks have been deposited in the Electron Microscopy Data Bank under accession codes EMDB-60459 [https://www.ebi.ac.uk/pdbe/entry/emdb/EMD-60459] (AtALMT9 sterol1 class), EMDB-60461 [https://www.ebi.ac.uk/pdbe/entry/emdb/EMD-60461] (AtALMT9 sterol2 class), EMDB-60462 [https://www.ebi.ac.uk/pdbe/entry/emdb/EMD-60462] (AtALMT9 N class), EMDB-60463 [https://www.ebi.ac.uk/pdbe/entry/emdb/EMD-60463] (AtALMT9 W class), EMDB-60464 [https://www.ebi.ac.uk/pdbe/entry/emdb/EMD-60464] (AtALMT9 cis1-PI/PIP class), EMDB-60465 [https://www.ebi.ac.uk/pdbe/entry/emdb/EMD-60465] (AtALMT9 cis2 class), EMDB-60466 [https://www.ebi.ac.uk/pdbe/entry/emdb/EMD-60466] (AtALMT9 intermediate class), EMDB-60467 [https://www.ebi.ac.uk/pdbe/entry/emdb/EMD-60467] (AtALMT9 trans1 class), EMDB-60468 [https://www.ebi.ac.uk/pdbe/entry/emdb/EMD-60468] (AtALMT9 trans2 class), and EMDB-61818 [https://www.ebi.ac.uk/pdbe/entry/emdb/EMD-61818] (AtALMT1). The molecular dynamics simulation files, including trajectories, structures, and parameters, have been deposited in the Zenodo under accession code 14177701 [https://doi.org/10.5281/zenodo.14177701]. The mass spectrometry data have been deposited in the ProteomeXchange Consortium via the PRIDE partner repository under accession code PXD059651 [https://www.ebi.ac.uk/pride/archive/projects/pxd059651]. The referenced coordinates have been deposited in the Protein Data Bank under accession codes 7W6K [http://doi.org/10.2210/pdb7w6k/pdb] (GmALMT12), 7VQ4 [http://doi.org/10.2210/pdb7vq4/pdb] (AtALMT1 apo / pH 7.5), 7VQ7 [http://doi.org/10.2210/pdb7vq7/pdb] (AtALMT1 Al^{3+} / pH 5.0), 8HIW [http://doi.org/10.2210/pdb8hiw/pdb] (AtALMT9 apo / pH 7.5) 8HIY [http://doi.org/10.2210/pdb8hiy/pdb] (AtALMT9 malate / pH 7.5), and 8ZVF [http://doi.org/10.2210/pdb8zvf/pdb] (AtALMT9 malate / pH 5.0). The source data underlying Figures 1g-i, 2j, k, 3b, d, e, 4c, i, j, l, m, and 5h, Supplementary Figures 1b, 8, 10b-g, 11b, d, f, h, j, 12d-f, 13a, c-f, h, i, 14b, and 20e are provided as a Source Data file.

Research involving human participants, their data, or biological material

Policy information about studies with human participants or human data. See also policy information about sex, gender (identity/presentation), and sexual orientation and race, ethnicity and racism.

Reporting on sex and gender	N/A
Reporting on race, ethnicity, or other socially relevant groupings	N/A
Population characteristics	N/A
Recruitment	N/A
Ethics oversight	N/A

Note that full information on the approval of the study protocol must also be provided in the manuscript.

Field-specific reporting

Please select the one below that is the best fit for your research. If you are not sure, read the appropriate sections before making your selection.

☒ Life sciences ☐ Behavioural & social sciences ☐ Ecological, evolutionary & environmental sciences

For a reference copy of the document with all sections, see nature.com/documents/nr-reporting-summary-flat.pdf

Life sciences study design

All studies must disclose on these points even when the disclosure is negative.

Sample size	All Experiments were repeated as much as possible, at least three times.
Data exclusions	No data were excluded from electrophysiological data. In cryo-EM, homogeneous particles with high resolution were only used to reconstruct map in refinement as described in data processing workflow.
Replication	All electrophysiological experiments were repeated at least three times, and the number of replicates is indicated in figures.
Randomization	Randomizations of experiments was not relevant to this study, and all measurements were independent from others.
Blinding	Blinding experiments were not relevant to this study.

Reporting for specific materials, systems and methods

We require information from authors about some types of materials, experimental systems and methods used in many studies. Here, indicate whether each material, system or method listed is relevant to your study. If you are not sure if a list item applies to your research, read the appropriate section before selecting a response.

Materials & experimental systems

n/a	Involved in the study	
☒	☐	Antibodies
☐	☒	Eukaryotic cell lines
☒	☐	Palaeontology and archaeology
☒	☐	Animals and other organisms
☒	☐	Clinical data
☒	☐	Dual use research of concern
☐	☒	Plants

Methods

n/a	Involved in the study	
☒	☐	ChIP-seq
☒	☐	Flow cytometry
☒	☐	MRI-based neuroimaging

Eukaryotic cell lines

Policy information about cell lines and Sex and Gender in Research

Cell line source(s)	Sf9 (Gibco)
Authentication	Cell lines were authenticated from the distributors as described above.
Mycoplasma contamination	Cell lines were not tested for mycoplasma contamination.
Commonly misidentified lines (See ICLAC register)	N/A

Dual use research of concern

Policy information about dual use research of concern

Hazards

Could the accidental, deliberate or reckless misuse of agents or technologies generated in the work, or the application of information presented in the manuscript, pose a threat to:

No	Yes	
x		Public health
x		National security
x		Crops and/or livestock
x		Ecosystems
x		Any other significant area

Experiments of concern

Does the work involve any of these experiments of concern:

No	Yes	
x		Demonstrate how to render a vaccine ineffective
x		Confer resistance to therapeutically useful antibiotics or antiviral agents
x		Enhance the virulence of a pathogen or render a nonpathogen virulent
x		Increase transmissibility of a pathogen
x		Alter the host range of a pathogen
x		Enable evasion of diagnostic/detection modalities
x		Enable the weaponization of a biological agent or toxin
x		Any other potentially harmful combination of experiments and agents

Plants

Seed stocks	Nicotiana benthamiana seed stocks are amplified at the host institute (IPSiM, Montpellier)
Novel plant genotypes	N/A
Authentication	N/A
